# A Simple Framework for Agent-Based Modeling with Extracellular Matrix

**DOI:** 10.1007/s11538-024-01408-8

**Published:** 2025-02-12

**Authors:** John Metzcar, Ben S. Duggan, Brandon Fischer, Matthew Murphy, Randy Heiland, Paul Macklin

**Affiliations:** 1 Intelligent Systems Engineering, Indiana University, 700 N. Woodlawn, Bloomington, IN 47408 USA; 2 Informatics, Indiana University, 901 E. Tenth Street, Bloomington, IN 47408 USA; 3 Computer Science, Indiana University, 700 N. Woodlawn, Bloomington, IN 47408 USA

**Keywords:** Extracellular matrix, Agent-based modeling, Collective migration, Stigmergy, Fibrosis, Cancer and basement membrane invasion

## Abstract

Extracellular matrix (ECM) is a key component of the cellular microenvironment and critical in multiple disease and developmental processes. Representing ECM and cell-ECM interactions is a challenging multiscale problem as they span molecular-level details to tissue-level dynamics. While several computational frameworks exist for ECM modeling, they often focus on very detailed modeling of individual ECM fibers or represent only a single aspect of the ECM. Using the PhysiCell agent-based modeling platform, we developed a framework of intermediate detail with the ability to capture bidirectional cell-ECM interactions. We represent a small region of ECM, an ECM element, with three variables describing its local microstructure: anisotropy, density, and overall fiber orientation. To spatially model the ECM, we use an array of ECM elements. Cells remodel local ECM microstructure and in turn, local microstructure impacts cellular motility. We demonstrate the utility of this framework and reusability of its core cell-ECM interaction model through examples in cellular invasion, wound healing, basement membrane degradation, and leader-follower collective migration. Despite the relative simplicity of the framework, it is able to capture a broad range of cell-ECM interactions of interest to the modeling community. Furthermore, variables representing the ECM microstructure are accessible through simple programming interfaces. This allows them to impact cell behaviors, such as proliferation and death, without requiring custom code for each interaction, particularly through PhysiCell’s modeling grammar, enabling rapid modeling of a diverse range of cell-matrix biology. We make this framework available as a free and open source software package at https://github.com/PhysiCell-Models/collective-invasion.

**Supplementary Information:**

The online version contains supplementary material available at 10.1007/s11538-024-01408-8.

## Introduction

Extracellular matrix (ECM), the material in which cells live, plays a role in a host of biological processes, from facilitating cell-cell communication to tissue development and organization. It is diverse with composition-dependent biomechanical and biochemical properties that vary greatly across locations within the body (Frantz et al. 2010). It is a living material, actively maintained and modified by cells such as fibroblasts in wound healing and cancer cells in local cellular invasion (Frantz et al. 2010; Yue 2014; Dzobo and Dandara 2023). Its properties are formed from multiple interacting fibers, for example, through chemical crosslinking of ECM components (Yue 2014). These fibers are composed of proteins and polysaccharides which are typically organized into several large families: proteoglycans, laminins, fibronectin, and fibrous proteins, each of which can be further grouped by physical and chemical properties (Frantz et al. 2010). Fibrous proteins include the collagens, the primary structural component of interstitial ECM, and elastin, which provides elastic properties to the ECM. Basement membrane ECM, which surrounds and separates functional units of tissue, is typically composed of collagens interwoven with laminins (Yue 2014). Additionally, the ECM includes signaling molecules and many attachment points for cell-ECM interaction (Yue 2014; Dzobo and Dandara 2023). While cells generate and maintain ECM, ECM also impacts cellular behavior. In particular, matrix density can hinder or enhance cell migration depending on cell type (Zaman et al. 2006; Charras and Sahai 2014). Additionally, ECM fiber orientations can guide cellular migration through contact guidance, in which cells follow ECM, reorienting their motion to travel parallel to ECM fibers (Werb et al. 1996; Ewald et al. 2008; Ramirez-San Juan et al. 2017; Winkler et al. 2020; Lu et al. 2011; Cheung and Ewald 2014; Thrivikraman et al. 2021). For example, Carey et al. observed that fiber alignment induced anisotropic cell morphodynamics and spatial probing, guiding them along the axis of alignment (Carey et al. 2016). Furthermore, ECM-based signaling, both through mechanosensing and cell receptor binding to ECM molecules, can impact cell cycle and death (Hastings et al. 2019). Cell differentiation can also be impacted by ECM (Humphrey et al. 2014). Finally, changes in ECM microstructure are associated with disease processes, in particular aspects of cancer progression, cancer metastasis, and scarring (Winkler et al. 2020; Lu et al. 2011; Nguyen-Ngoc et al. 2012; Wynn and Vannella 2016).

Given the importance of ECM in biological processes, there are multiple models and frameworks for exploring ECM and cell-matrix interactions across a range of contexts such as cancer, wound healing, and fibrosis (Metzcar et al. 2019; Jorgensen and Sanders 2016; Leonard-Duke et al. 2020; Crossley et al. 2024). To provide a brief overview of previous works, we place these efforts in four categories: discrete force-based, continuous force-based, ECM density, and multicomponent that have both ECM density and orientation.

Examples of the discrete force-based frameworks include Schlüter et al. and Noël et al. where migrating cells and individual matrix fibers are embodied as agents with direct forces dictating motility and realignment respectively (Schlüter et al. 2012; Noël et al. 2024). Macnamara et al. also adopt this paradigm and couple it with a model of blood vessel-cell interactions (Macnamara et al. 2020). Other representations calculate forces over a network of individual spring-like elements. Zhu et al. construct ECM from a hook-and-spring network and Tozluoğlu et al. model individual fiber filaments as hook-and-spring networks (Zhu and Mogilner 2016; Tozluoğlu et al. 2013). Tsingos et al. model a network of ECM fibers as a bead-spring chain model and couple this with a cellular-Potts agent-based model (Graner and Glazier 1992; Tsingos et al. 2023).

Continuous force-based models represent ECM as a continuously deformable material. Examples include van Oers et al., who use a finite element model of ECM coupled with a cellular-Potts model of cells and look at how stiffness and deformability of the ECM impact cell migration (van Oers et al. 2014). Ahmadzadeh et al. use a stress-strain relationship to deform fibers and incorporate cell adhesions to investigate cellular level motility and morphology (Ahmadzadeh et al. 2017).

ECM density approaches often use partial-differential equations (PDEs) to model ECM density. Anderson and Chaplain use a continuous method that leads to a hybrid discrete-continuous model to study the interplay between angiogenesis and ECM (Anderson and Chaplain 1998). Zeng et al. use a fibronectin concentration in developmental biology applications (Zeng et al. 2004). Daube et al. use a PDE for a scalar ECM density field in the context of sprouting angiogenesis in tumors (Daub and Merks 2013). Trucu et al. model invasion as a moving boundary problem with ECM represented as a scalar field (Trucu et al. 2013). Building off that work, Shuttleworth and Trucu add non-local effects as well as ECM orientation and degradation to the invasive boundary (Shuttleworth and Trucu 2019, 2020). Gonçalves and Garcia-Aznar represent the ECM as a density and use experimental migration data to explore tumor spheroids (Gonçalves and Garcia-Aznar 2021). Lastly, Ruscone et al. study cancer invasion using PhysiBoSS (Letort et al. 2019; Ponce-de-Leon et al. 2023), an implementation of PhysiCell (Ghaffarizadeh et al. 2018) with the MaBoSS Boolean network simulator (Stoll et al. 2017), producing a model incorporating intracellular signaling activated by contact with a bulk ECM (Ruscone et al. 2023).

Finally, there are models that explicitly represent fiber orientation or fiber orientation and density. Chauvier et al. and Hillen et al. use transport models to look at ECM (Chauviere et al. 2007; Hillen 2006). This work was extended by Painter to introduce a macroscopic ECM density and fiber orientation which was then applied to model tumor invasion (Painter 2009). Other authors model ECM directly as a phase in a cellular-Potts model exploring angiogenesis, tumor invasion, and impacts of cytoplasm and nucleus interaction on migration (Bauer et al. 2007; Rubenstein and Kaufman 2008; Scianna et al. 2013). Some authors also form long-range fibers by viewing sets of contiguous points occupied by ECM as fibers. They can then more directly represent fiber orientation and length in addition to density (Szabó et al. 2012; Kumar et al. 2016). Park et al. use a modified Vicsek (flocking) model (Vicsek et al. 1995) to explore contact inhibition of locomotion in the formation of heterogeneous ECM fiber orientations (Park et al. 2020). Suveges et al. use a hybrid agent-based model and two phase continuous-ECM field approach (oriented, fibrous phase and non-fibrous phase) to study collective migration on oriented ECM (Suveges et al. 2021). Dallon, Sherratt and collaborators have multiple studies on ECM that include a concept of density and orientation and focus primarily on wound healing and scar formation. The models can be split into hybrid models (discrete cells and continuous ECM) or fully continuous models. Examples of the continuous models include reaction-diffusion and integro-partial differential equation models of wound healing (Olsen et al. 1999; Dallon and Sherratt 2000). The hybrid-discrete continuous models include a model of ECM fiber reorientation, an extension to include ECM density, and a third extension to include the impacts of time varying transforming growth factor $\beta$ profiles on all models including ECM contact guidance (Dallon et al. 1999, 2000, 2001). For additional details on these foundational works in modeling the ECM and wound healing, see Sherrat and Dallon’s review (Sherratt and Dallon 2002). In a later paper, the model is further extended to combine chemotactic and ECM contact guidance, still in the context of wound healing (McDougall et al. 2006). Finally, in work similar to Dallon, Sherratt, and colleagues, Martinson et al. model a field of discrete ECM puncta (dots of immature proto-fibrous ECM) and neural crest cell-driven maturation and alignment of the puncta fibers. Using both number of ECM puncta and their orientations to alter cell motion, they study how these factors influence collective migration of neural crest cells (Martinson et al. 2023).

In this work, we draw on these prior modeling approaches to capture essential aspects of bidirectional, local cell-ECM interactions. We divide the ECM into volumetric elements which track overall local ECM density, fiber orientation, and fiber-fiber alignment (anisotropy), properties collectively referred to as the local microstructure. Individual cell agents can remodel each of these properties, while these properties can influence cell behaviors including migration speed, chemotactic response, motility direction, proliferation, death, secretion, and differentiation. This mesoscale approach spans between a coarser ECM density field and more fine-grained approaches that might represent each fiber. It yields a higher dimensional characterization of ECM than ECM density alone, allowing a more diverse class of modeling assumptions, but eliminates the complexity of discrete force-based frameworks and high-order PDE models (particularly those with tensorial terms), simplifying integration with a broader class of cell behaviors than in prior works. It opens the possibility of linking important aspects of cell biology such as secretion, phagocytosis, effector attack, differentiation, proliferation, death, and cell fusion to this higher-dimensional set of ECM properties as they vary both spatially and over time. We implement this as a framework that can be readily used to incorporate local ECM effects into agent-based models, pairing it with the open source package PhysiCell (Ghaffarizadeh et al. 2018). To demonstrate the versatility of the framework, we present four sample models focused on cell migration and local degradation, deposition, and reorientation of matrix. In the first example, to test the framework’s ability to capture the complex effects of prior models, we replicate aspects of a study from Painter in which a front of tumor cells invade different scenarios of ECM configurations (Painter 2009). For the second example, we model wound-related fibrosis where activated fibroblasts generate a region of high ECM density that cells cannot enter or leave. In the third, recruited fibroblasts degrade a basement membrane and allow a tumor to progress from locally confined to local invasion. The final example is a model of collective migration which can produce both stigmergy (indirect coordination of migration through changes in the environment) and leader-follower migration, with more aggressive leader cells enabling mixed clusters of cells to invade surrounding tissue. Overall, our framework builds on previous work and is especially inspired by Dallon et al. (1999), who developed a model of cell-ECM interactions focused on wound healing. We generalized a similar model into an extensible framework that can be rapidly adapted to new problems, in particular through use of emerging rules-based formulations (Johnson et al. 2023).

We present our work as follows: Section 2 introduces the ECM representation and cell-ECM interactions and implementation details. Sections 3.1, 3.2, 3.3, 3.4 present the example models. Section 4 discusses findings, limitations, and future directions for the framework. Our supplementary material covers the cell-ECM interaction model in detail as well as details of cell-cell and cell-environment mechanics (Ghaffarizadeh et al. 2018), PhysiCell rules (Johnson et al. 2023), and example model parameters.

## Methods: Framework Description

In this section, we first provide the conceptual background and formulation of the ECM and cell-ECM interactions framework (the ECM model). Then, we illustrate the framework with a series of computational results highlighting important aspects of the framework. Finally, we give an overview of the computational details.

### ECM and cell-ECM Interaction Models

To enable explorations of ECM-mediated cellular communication and interactions, we developed a three variable ECM representation (orientation, anisotropy and density) and interaction rules for cells and the ECM. We use an agent-based approach to represent cells and a continuum model to represent the fine structure of ECM. Broadly, individual ECM elements are locally remodeled, or modified, by cell migration and ECM production and degradation and cell motility, proliferation, death, and other behaviors can be impacted by ECM element variables.

#### Extracellular Matrix Representation

**Fig. 1:**
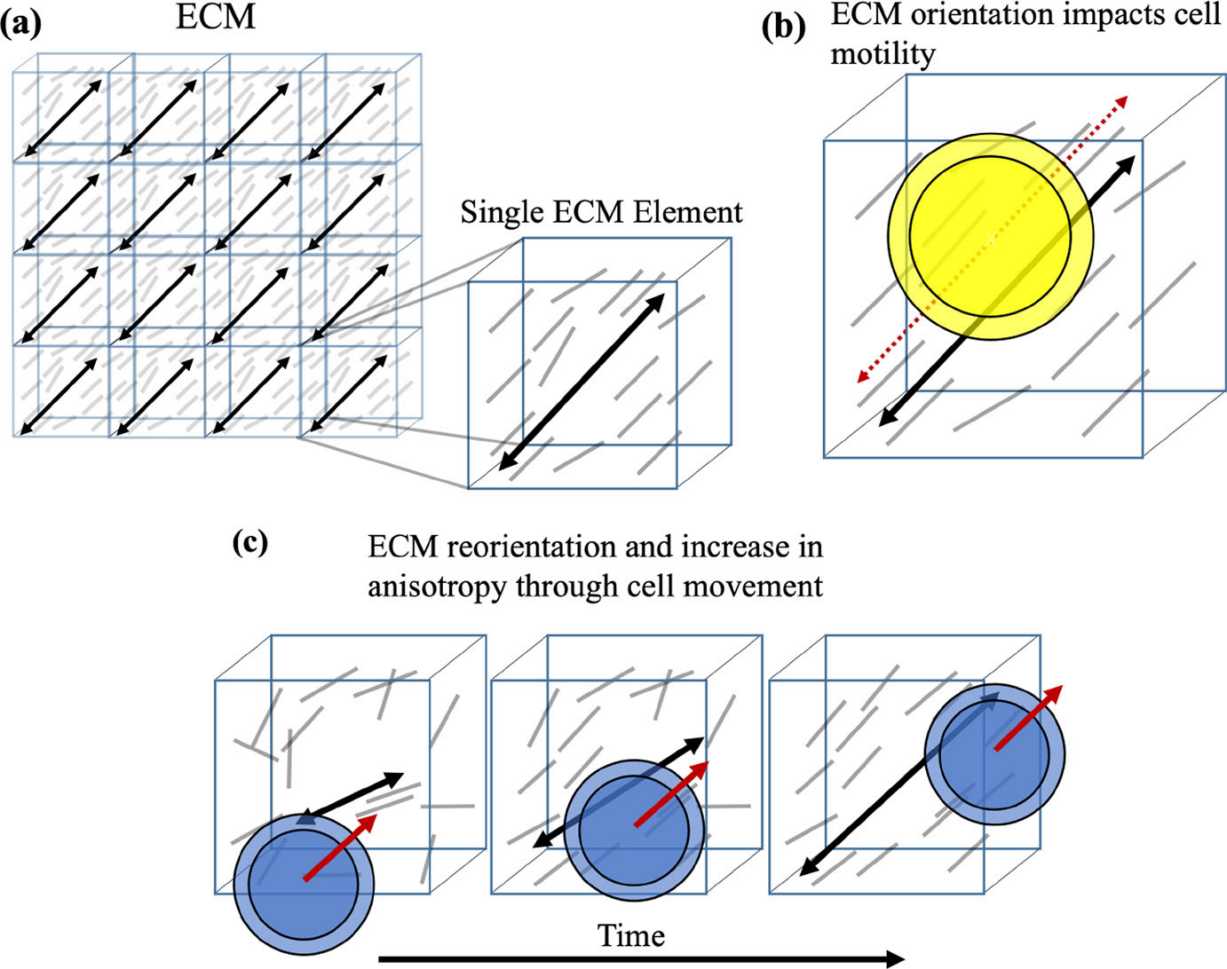
Schematic of ECM model and motility-based cell-ECM interactions: **a** Illustration of ECM, composed of tiled ECM elements. We conceive of an element of ECM as containing many fibers. However, rather than modeling each of those fibers, we instead model the ensemble, or mean, properties of the set of fibers in a given volume. We show a schematic of a single element containing many fibers (grey line segments, for illustration purposes only) which we quantify by an overall (average) fiber orientation and anisotropy (amount of self-alignment), shown by the black double-headed arrow and its length. **b** An element’s overall fiber orientation impacts a cell’s motility, providing a preferred angle of travel through the element (dashed red arrow). The degree of influence is related to the amount of anisotropy and the cell’s ability to sense and respond to the ECM cues. **c** Cell movement (cell direction - red arrow) remodels ECM by aligning its element’s orientation and anisotropy. Cells align an element’s orientation parallel to their direction of travel and increase anisotropy proportional to cell speed (Color figure online)

Our model is intended to capture key aspects of a small region, or element, of ECM. We conceive of an element of ECM as having three variables: an average fiber orientation (*orientation*), a relative local fiber-fiber alignment correlation (*anisotropy*), and a quantity that captures the relative amount of ECM material present in an element (*density*). Assembling many of these elements together makes a whole ECM. Figure 1a shows an ECM, which is comprised of tiled ECM elements. The inset shows a single element. It is conceived of as containing many fibers (for illustrative purposes, shown as grey line segments). However, the individual fibers are unmodeled and instead an ensemble of fibers are represented through an average orientation and anisotropy (the black double-headed arrow and its length) and volumetric density (illustrated as number of fibers per element in the schematic).

Mathematically, we center an element at a location $\vec {x}$ in space and define the element variables as follows:

- ***Orientation:*** $\vec {f}(\vec {x}$), the average local fiber direction, represented as a unit length three-dimensional vector
- ***Anisotropy:*** $a(\vec {x}$), average local fiber-fiber alignment correlation (range 0 - 1). At zero, there is no correlation and at one, locally there is complete fiber-to-fiber correlation
- ***Density:*** $\rho$($\vec {x}$), volume fraction of fibers, which we will refer to as the average local fiber density (range 0 - 1). Zero refers to complete absence of fibers and one to completely packed with fibers without void space.

Without a loss of generality, for our simulations and implementation, we arrange the elements in a Cartesian grid.

#### Cell-Based Modeling Framework

We use the PhysiCell tissue modeling system as our agent-based modeling frameworkto implement the ECM and cell-ECM interactions models (Ghaffarizadeh et al. 2018; Johnson et al. 2023). PhysiCell uses a center-based, off-lattice representation of cells and implements physical cell-cell mechanics. It takes a phenotypic approach to encoding cell behaviors withletting each cell-agent carrying its own parameters and behaviors. Additional details of the mechanics and rules used in PhysiCell are in Supplementary Material Sections 3 and 4 respectively. To model the diffusive microenvironment, we use BioFVM, which comes with PhysiCell. BioFVM is a diffusion solver that uses a Cartesian representation of space and finite volume method to efficiently handle multiple diffusing substrates over tissue-scale sized domains (Ghaffarizadeh et al. 2016).

#### Cell-ECM Interactions

We also model interactions between cells and the ECM. The conceptual model is below with the detailed mathematical model provided in Supplementary Material Sections 1, 2, and 4.

- *ECM impacts cell migration (Fig.* 1*b):*
  - Fiber orientation provides directional cues (Werb et al. 1996; Ewald et al. 2008; Ramirez-San Juan et al. 2017; Winkler et al. 2020; Lu et al. 2011; Cheung and Ewald 2014; Thrivikraman et al. 2021).
  - Anisotropy gives a strength of directional cue: high anisotropy increases an ECM element’s influence on direction of cell migration (Thrivikraman et al. 2021).
  - Density influences cell speed: too little ECM, cells have nothing to attach to; too much, cells cannot pass (Zaman et al. 2006; Charras and Sahai 2014).
- *Cell migration and movement impact microstructure (Fig.* 1*c):*
  - Direction of cell migration reorients an ECM elements’s orientation (Frantz et al. 2010; Yue 2014; Dzobo and Dandara 2023).
  - Cell-ECM element contact increases ECM anisotropy proportional to cell speed (Frantz et al. 2010; Yue 2014; Dzobo and Dandara 2023).
  - Cells remodel ECM density towards a target value (Frantz et al. 2010; Yue 2014; Dzobo and Dandara 2023).

This model is motivated by findings in the developmental, disease, and tumor biology literature as well as inspired by previous modeling efforts (Frantz et al. 2010; Lu et al. 2011; Cheung et al. 2013; Winkler et al. 2020; Dallon et al. 1999). The cell-ECM interactions are specified at the cellular level, enabling a variety of cell-ECM interactions, in particular changes in cell motility and ECM remodeling capabilities (Werb et al. 1996; Lu et al. 2011; Cheung et al. 2013). Additionally, ECM variables can be used to impact other cellular behaviors such as proliferation and death. Finally, we note and will demonstrate that these features can be integrated with others such as sensitivity to chemical cues and cell-cell adhesion to obtain an even richer range of cell behaviors.

### Individual Features of the Cell-ECM Interaction Model

In this proof of concept section, results isolate and demonstrate a single or small number of aspects of the cell-ECM interaction model presented in Sect. 2.1. To aid in isolating only ECM-to-cell and cell-to-ECM effects, we disabled cell-cell mechanical interactions by setting cell-cell adhesion and repulsion ($C_{\text {cca}}$ and $C_{\text {ccr}}$ in Supplementary Material Equations 22 and 24 respectively) to zero. We used multiple cells per result to highlight the uniformity of interactions across the computational domain and as a visual aid rather than to explore how cell-cell interactions may impact the simulations.

#### Cells Alter Microstructure, Producing Directional Signals in the ECM

***Combing the ECM: Local ECM fiber orientation and alignment remodeling produces extended paths in the ECM***

**Fig. 2:**
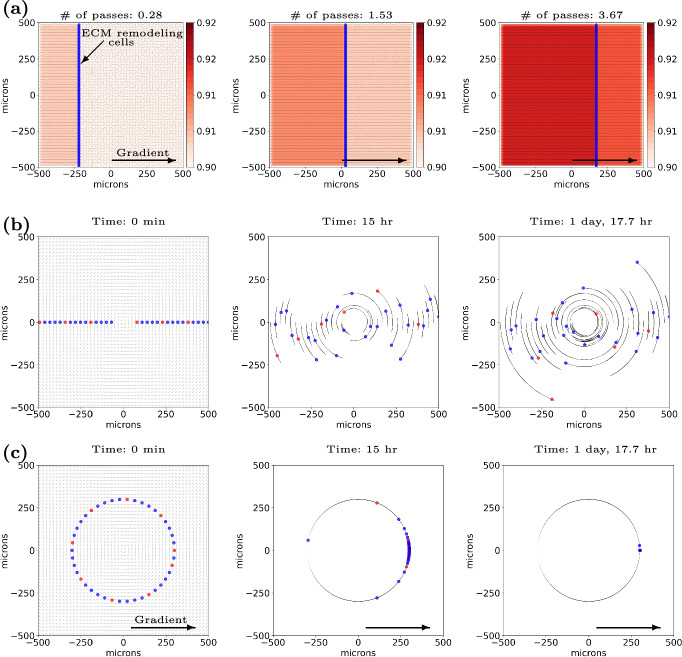
Highlights of individual aspects of the cell-ECM interactions: **a - Modifying ECM microstructure** A line of ECM remodeling cells (blue circles), driven by a constant chemical gradient (black arrow), move in the positive x-direction, reorienting the initially randomly oriented ECM element orientations (black line segments) (left-most image in **a**. Upon reaching the simulation boundary, the cells reset to the opposite boundary, and remodeling continues (middle - 1.53 passes). At four passes (left region of the right-most image in **a**, each ECM element is almost completely reoriented parallel to the direction of travel of the cells and anisotropy has increased from 0.90 to $\sim$0.92. Note, we run this in 2-D to aid in visualization and demonstrate uniformity across the domain. **b, c - Following ECM**. Both simulations use ECM elements that are highly anisotropic (*a* uniformly set to 1.0) and element orientations arranged to form approximate concentric circles. The simulation in **b** begins with ECM sensitive cells almost equally spaced along the line y=0 (leftmost image - cells shown in red and blue; color is for visual contrast only). Cells follow the ECM circles in an approximate 1-D random walk due to ECM following and the lack of an external driving direction (*e.g.* chemotaxis), forming circular arcs (small black arrows marking cell position history every 6 minutes). The piecewise linear representation of the circular ECM tracks are more accurate as distance from the center increases (and curvature decreases). Because of this, cells following the farther piecewise linear tracks stay closer to the theoretical circular paths. The simulation in **c** contrasts with **b** by providing an additional environmental cue - a chemical gradient. As the ECM sensitive cells integrate both signals, in contrast to **b**, their motion is no longer random and they travel uniformly in the direction of the gradient. With a circular initial configuration (left), the cells follow each other along the same circle (middle and right), coming to a point of relative stability where the ECM orientation is exactly perpendicular to the chemical field. Note that the ECM is static in **b** and **c** and does not vary across time. Videos are available. See Supplementary Material.

Remodeling of the ECM elements can produce paths that ECM sensitive cells can then follow. In Fig. 2a, a column of cells move deterministically from left to right across a field of un-oriented ECM aligning it in the direction of travel. Each ECM element is initialized with a random orientation and anisotropy of 0.9. Remodeling cells move across the domain, producing individual changes in ECM elements as they travel. After reaching the simulation boundary, the cells reset to the opposite boundary, and remodeling continues. In this case, after four remodeling passes (transits) through the matrix, the ECM is aligned almost completely parallel to the direction of travel with the additional effect of a slight increase in anisotropy, demonstrating ECM reorientation, and producing oriented paths across the domain. Note that we run this simulation in 2-D to aid in visualization and to demonstrate uniformity across the domain. A video is available in the Supplementary Material.

#### Cells Read Signals in the ECM, Impacting Their Migration

All results in this section were produced with anisotropy set uniformly to 1.0, the maximum and most impactful value, and density uniformly set to 0.5, the value producing maximum cell speed ($\rho _{\text {ideal}}$ from Supplementary Material Equation 9). Additionally, the chemical and physical (ECM) environments are static in these cases, meaning no changes in chemical substrate values or ECM remodeling occurs. This ensures that only dynamics between a cell and the ECM or respectively among a cell, ECM, and chemical field impact the results. In this set of simulations, the cells are highly sensitive to ECM (*s* = 1.0 from Supplementary Material Equation 9) as well as to the chemical gradient (*b* = 1.0 from Supplementary Material Equation 15) when present.

***Cells can closely follow paths in the ECM***

Supplementary Material Figure 3 shows influence of ECM on cell migration (ECM contact guidance) combined with a chemotactic field going to the right. The leftmost subfigure shows the initial cell positions as well as the ECM element fiber orientations (45$^\circ$ and -45$^\circ$ from the x-axis in the top and bottom sections of the domain respectively). Without ECM influence, the cells would simply move to the right. Instead, the ECM biases the cell paths away from the path set by the gradient. The middle and rightmost images include the cell’s positional history, shown as arrows trailing the blue and red cells (note in this and in all images in this section, the cell color is simply for visual contrast). A video is available in the Supplementary Material.

***Lacking additional directional cues, cells perform approximate 1-D random motion along ECM paths***

Fig. 2b shows the results of cell motion on circular ECM. We show the initial cell configuration as well as the ECM element orientations on the left with the middle and right images including cell positional history at 15 hours and 41.7 hours simulated time respectively. Our ECM model does not encode a preferred direction. Instead, without an externally set direction (*e.g.* a chemotactic direction), cells select a random direction (see the end of Supplementary Material Section 1). However, contact guidance limits cell motion to be along the circular ECM paths. Thus, we observe approximate 1-D random motion along the ECM circles in this proof of concept simulation. Because of the curved tracks and error due to ECM discretization in highly curved regions (center of domain in this case), contrasting with Supplementary Figure 3, some cells move off their initial paths out to ECM circles of higher radius. We expand on this phenomenon in the discussion (Section 4). Due to the high impacts of ECM discretization at the center of the domain, for this simulation, we exclude the central region with radius of 80 $\mu \text {m}$ or less. A video is available in the Supplementary Material.

***Cells can integrate environmental signals and follow non-linear paths***

Fig. 2c shows the results of cell motion transiting circular ECM with an additional cue supplied by a chemical gradient. This additional information causes the cells to follow the ECM circle on which they were initially placed to the right (the direction of the chemical gradient). Note that at locations where the chemical gradient and ECM orientation are perpendicular, cells are in a quasi-stable state as the two directional cues cancel. This meta-stable state is seen in the lagging cell in the middle plot of Fig. 2c. Note that all other cells in this plot, except the lagging blue cell on the left side of the image, have moved many cell lengths from their initial positions. This cell began at the x-axis, which defines a line in which all units of ECM have an orientation perpendicular to the chemical field. Likewise, all the cells converge at the x-axis, where their initial ECM contour becomes perpendicular to the chemical field. Unlike in Fig. 2b, with cells placed further from the origin and in a region of lower curvature, we do not observe “track jumping". A video is linked in the Supplementary Material Section 6.

### Computational Method Details and Data Availability

This work is implemented and run in PhysiCell 1.12.0 (Ghaffarizadeh et al. 2018). It also uses the recently introduced PhysiCell rules (Johnson et al. 2023). All code is available with a permissive BSD license, including the ECM extensions and sample models, at GitHub with the latest release located here. The code is cross-platform capable and has been successfully compiled and executed on Ubuntu 22.04, Windows Server 2022, and MacOS 12. All simulation data for this paper is available for download at Zenodo. A free, interactive cloud-hosted version of the leader-follower model (see Section 3.4) is available at nanoHUB (note - sign up is required).

On consumer-grade hardware (2019 Macbook Pro), typical wall times were 13 minutes for the longer fibrosis simulation and 10 minutes for the invasive carcinoma and leader-follower simulations. The invasive cellular front had typical wall times of 3-4 minutes. On high-performance computing hardware, mean wall times were 4 minutes for the fibrosis simulation, 3.2 minutes for the basement membrane degradation and leader-follower collective migration simulations, and 1 minute for the invasive cellular front. The simulations start with 412 (fibrosis), 517 (basement membrane degradation) and 703 (leader-follower) cells and simulate 15 days, 10 days, and 10 days respectively. The fibrosis model has a decreasing number agents as the simulation progresses but also simulates 50% more time, hence its longer run time. The invasive cellular front starts with 30 cells, building to 900 cells when the simulations completes at 5 days.

We performed initial model exploration using DAPT 0.9.2. DAPT is a straightforward way to organize model exploration on a mixed compute resources for small teams. For more information see Duggan et al. (2021).

We used xml2jupyter to transform this command line C++ code into a Jupyter notebook-based graphical user interface (Heiland et al. 2019). The resulting interface is deployed at nanoHUB.

## Framework Examples: Invasive Cellular Front, Fibrosis, Basement Membrane Degradation, and Collective Migration

We present four modeling vignettes to demonstrate framework features and ability to represent a range of biological phenomena, with a focus on ECM remodeling and cell motility. To help isolate dynamics due to physical interactions and cell motility, the presented examples do not include cell proliferation and death (except where required) as features. We use the default cell definition from PhysiCell, varying relevant parameters and behaviors as noted in each example. To examine the robustness of the simulations to stochastic variation, we ran each primary model 21 times and include a representative example for each modeling vignette.

For the chemical microenvironment, we use default settings unless noted otherwise. We pre-condition the domain by running the diffusion solver for 10 simulated minutes before starting the simulation. For ECM, we use the model described in Section 2.1. We place the ECM elements overlaying the BioFVM voxels exactly; the two meshes have a shared origin and coordinate system. See each example for exact microenvironment set up.

Finally, we note that the models were developed as 2-D simulations with cells confined to a single layer of 3-D voxels.

### Invasive Cellular Front Pushing into ECM

As a test of this framework’s ability to express prior complex models, we reproduced aspects of Painter’s 2009 computational study (Painter 2009), in particular the first example: a model of an expanding tumor pushing cells into surrounding, structured ECM, as illustrated in Figure 10 of Painter 2009. In that work, a constant influx of cells was introduced on a domain boundary, with cells exhibiting strong ECM contact guidance. The ECM orientations were either random, parallel to the domain edge, perpendicular to the domain edge, or a mix of parallel and perpendicular orientation. The work found that randomly and perpendicularly oriented matrices permitted faster invasion compared to the parallel setting and that the mixed setting produced a heterogenous pattern of invasion, with fastest invasion where the ECM was perpendicular to the interface. We tested whether the framework could be adapted to these scenarios and qualitatively reproduce their findings.

To emulate these scenarios in our agent-based system, we introduced a constant flux of cells (instantiate 30 new cells every 180 minutes along domain edge) at the bottom edge of a 600 $\mu \text {m}$ by 1000 $\mu \text {m}$ domain and simulated for 5 days (final cell count of 900). The cells had cell-cell adhesion ($C_{\text {cca}}$) and repulsion strength ($C_{\text {ccr}}$) of 0.4 and 25.0 $\frac{\mu \text {m}}{\text {min}}$ respectively, base speed $S_{\text {max}}$ of 1.25 $\frac{\mu \text {m}}{\text {min}}$, and ECM sensitivity, *s*, of 1.0. The cells were set to not remodel ECM (ECM is static) and the ECM was highly anisotropic (*a* = 1.0 throughout domain). In this scenario, cell-cell repulsion and base cell speed are increased relative to the default values of 10 $\frac{\mu \text {m}}{\text {min}}$ and 1.0 $\frac{\mu \text {m}}{\text {min}}$. For additional parameter and simulation details, see Supplementary Material (Section 5.1). See Supplementary Material Section 3 for details on the cell-cell adhesion model. We quantified the speed of the invasive front through histograms of cell positions in the y-axis (40 bins of 25 $\mu \text {m}$ each) then determined the histogram bin containing the 95th percentile of cell count at five simulated days. We produced stochastic replicates for each scenario.

**Fig. 3:**
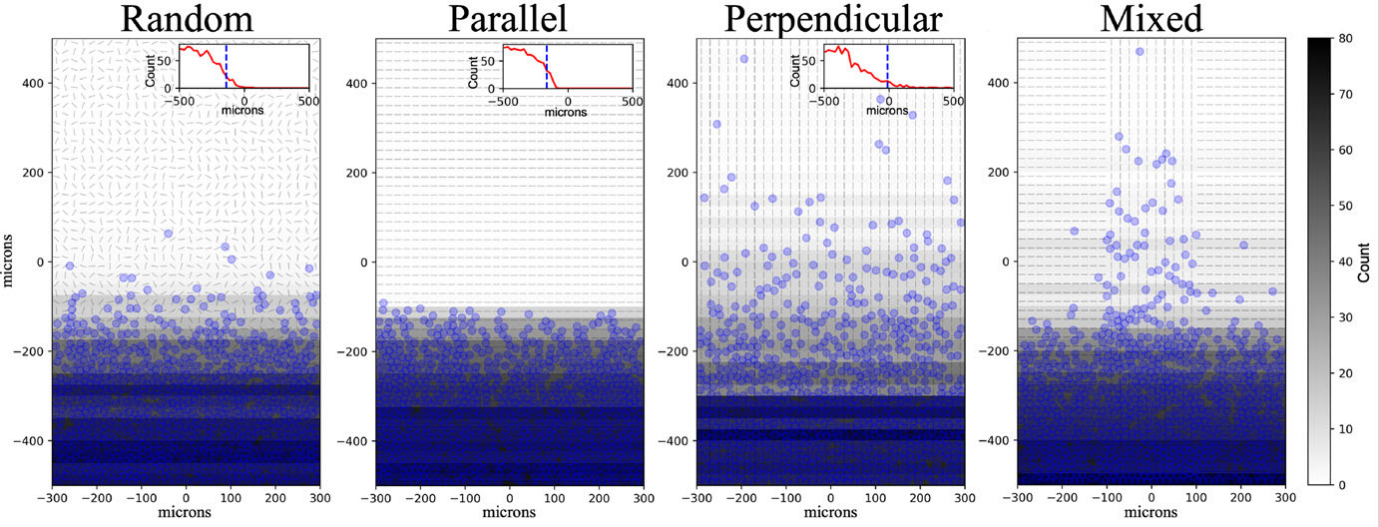
Invasive cellular front pushing into ECM: To reproduce aspects of Painter 2009’s first modeling example of an expanding tumor (Painter 2009), we produced four ECM orientation configurations - random, parallel to advancing front, perpendicular to advancing front, and the mixed case of both parallel and perpendicular. In all cases, the ECM was static (no ECM remodeling) and cells entered the simulation at the bottom edge of the domain, moving with random, contact-guided motility. 30 cells were introduced every 180 minutes with no flux conditions at the boundaries. We show each simulation at five days simulated time. To assess cell penetration into the domain, we produce histograms of cell positions in the y-axis (40 bins of 25 $\mu \text {m}$ each). The horizontal contours are shaded according to the histogram bin counts. The insets in the upper right of the random, parallel, and perpendicular conditions show the binned cell counts viewed in profile. The dashed blue line indicates the center of the strip where 95% of total cell count is passed. ECM orientation shown in grey line segments.

Consistent with the work by Painter, cells traveled further in matrices with perpendicular and random ECM orientations compared to the parallel setting. Over the 21 replicates, the fastest advances of the invasive front occurred when the ECM orientations were perpendicular to the front followed by the random orientations and finally parallel orientations. Summary statistics and visual comparison across replicates are shown in the Supplementary Materials (Supplementary Table 19 and Supplementary Figure 4). Figure 3 shows representative examples of the stochastic replicates we conducted. The changes in the invasive front across scenarios can be seen in both the deeper penetration of cells into the domain in the perpendicular and random cases as well as the cell count contours (black-white horizontal bars) in Fig. 3. Furthermore, the cell number profiles (insets in upper right of the random, parallel, and perpendicular ECM scenario plots) also show differences. In the more restrictive case of parallel ECM, a relatively sharp boundary is formed, as seen in the changing slope of the cell count profile, compared to the more shallow slope of the profiles in the random and perpendicular cases. Additionally, the random orientation gives front shapes similar to a diffusive front (e.g., similar to those seen in Fisher’s equations Sherratt and Chaplain 2001), whereas the scenario with perpendicular orientation spreads faster suggestive of superdiffusive phenomena (Jiang et al. 2014; Volpert et al. 2010). Finally, in the mixed orientation scenario, we see that invasion was fastest in the region with orientations perpendicular to the interface and slowest in the regions of parallel orientations, matching Painter 2009 (Painter 2009). Videos available in the Supplementary Material and full stochastic replicates available in the data supplement at Zenodo.

### Wound Healing: ECM Deposition, Fibrosis, and Enclosure of a Wound

We produced a simple model of fibrosis. We model the recruitment of cells to clear an injury - first macrophages and subsequent recruitment of fibroblasts to rebuild the ECM in the area of the injury (Gurtner et al. 2008; Wynn and Vannella 2016; Betensley et al. 2017). Fibroblasts are activated in the presence of macrophages, depositing ECM, leading to formation of a circle of very high ECM density and subsequent exclusion of cells from the wounded area.

**Fig. 4:**
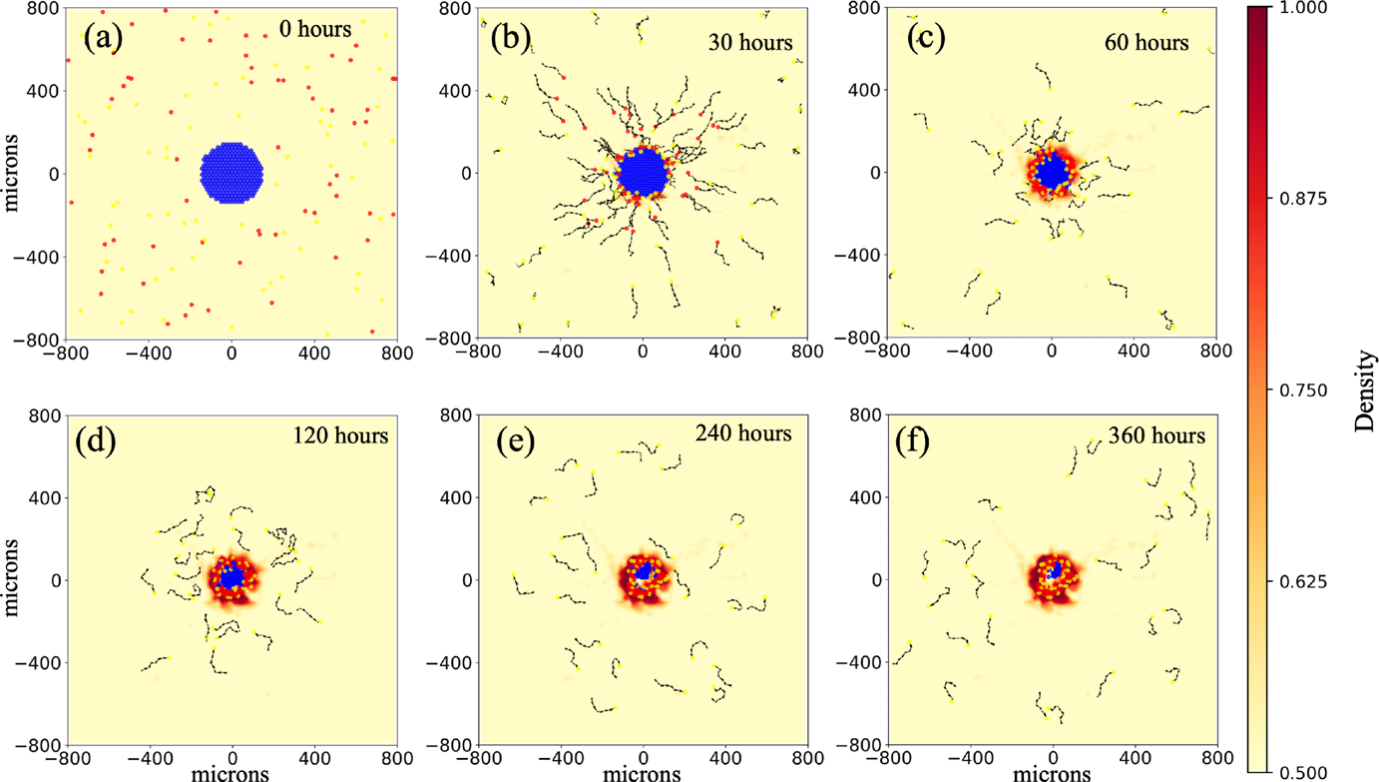
Fibroblast ECM secretion leads to cyst formation: Tissue injury, modeled as sudden death of a number of cells (blue cells) attracts macrophages (red) to remove dead cells (**a, b**). The macrophages recruit fibroblasts (yellow), which secrete ECM as part of the tissue repair process (**c, d**). However, prolonged contact with macrophages leads to continued secretion of ECM. This produces a region of impenetrable ECM which both traps the macrophages and fibroblasts and leads to a region that is relatively cell-free as the dead cells continue in their death process (**e, f**). Initial configuration - dead cells 175 $\mu \text {m}$ circle with macrophages and fibroblasts randomly placed outside of the distressed region. Yellow cells: fibroblasts. Red cells: macrophages. Blue cells: distressed/dead cells. Yellow-to-red contour plot: ECM density. Black arrows: cell positional history, going back eight time points (sampled every 30 minutes). This is available as a video. See Supplementary Material (Color figure online)

*Model results and cellular behaviors* We use three cell types - distressed/dead cells, macrophages, and fibroblasts similarly to Cumming et al. (2009). The key rules of the model are summarized in Table 1. The distressed cells die immediately and release debris after having already begun to release debris prior to dying (Fig. 4a). This is similar to an injury or some other insult that could cause a small region of cells to die at once. The debris recruit macrophages (shown in red) which chemotax up the debris gradient (not shown). Upon reaching the damaged cells, macrophages phagocytose the dead cells and also send out an inflammatory signal to initiate additional tissue repair. This signal recruits fibroblasts (yellow) via chemotaxis, which attempt to repair the damaged tissue by secreting ECM materials and increase density (yellow-to-red filled contours) above $\rho _{\text {ideal}}$, the ECM density of maximum cell speed (Fig. 4b,c,d). As fibroblasts and macrophages remain in contact, ECM continues to be produced. This eventually leads to the entrapment of the recruited cells. As the dead cells continue to shrink due to their death process, a cyst-like relatively cell- and ECM-free region forms, enclosed within a circle of impenetrable ECM (Fig. 4e,f). See Supplementary Material Table 7 for additional details. A video of results presented in Figure 4 is available in the Supplementary Material.

Of the 21 stochastic replicates, 15 generated a completely enclosed region (such as the representative example in Fig. 4) and six resulted in a partially enclosed region. See the simulation data set at Zenodo for individual results.

**Table 1:** Fibrosis model: principle behaviors, parameters, and systems effects. See Supplementary Material for parameter definitions

Cell type	Behavior	Key parameter(s)	Key effects
Distressed cells	Secrete debris	Debris secretion rate	Recruits macrophages to dead cells
	Death	NA - cells die immediately at start of simulation	Begins fibrotic cascade
Macrophages	Chemotaxis up debris gradients	Bias	Enables macrophages to find dead cells
	ECM density response	$\rho _{\text {l}}$, $\rho _{\text {ideal}}$, $\rho _{\text {h}}$	Cell speed is reduced as ECM density moves away from ideal value (0.5)
	Consume debris	Debris uptake rate	Aids in generating gradient
	Consume dead cells	Dead phagocytosis rate, phagocytosis rule saturation value and half-maximum	Enables clearance of cells
	Secretion of inflammatory signal	Signal rule saturation and half-maximum values	Recruits fibroblasts to region of damaged cells
Fibroblasts	Chemotaxis up inflammatory gradients	Bias	Enables fibroblasts to find macrophages
	ECM density response	$\rho _{\text {l}}$, $\rho _{\text {ideal}}$, $\rho _{\text {h}}$	Cell speed is reduced as ECM density moves away from ideal value (0.5)
	Degrading/depositing ECM density	ECM density target value, ECM density secretion rule saturation, and half-maximum values	Can trap cells if deposited at high enough amount

*Tissue microenvironment* We use two diffusive chemical fields in this simulation. Debris is emitted by distressed and dead cells and has a very ssmall diffusion coefficient and no decay, representing the slow movement of cellular debris and lack of decomposition on the simulated time scales (macrophage consumption is the only removal process). This field represents the materials that distressed and dead cells release as part of the death process. We also simulate an inflammatory signal, released by macrophages as part of their response to contact with dead cells. It has a characteristic length scale of 32 $\mu \text {m}$, or approximately 2.5 cell diameters. Both fields have no flux conditions.

The ECM density begins uniform with a value of 0.5 throughout the domain. Fibroblasts modify the density when recruited by macrophages. We note that in this simulation, only ECM density impacts cell motility, as the motile cell populations are modeled as being insensitive to ECM orientation cues.

See Supplementary Material Tables 8 and 9 for additional details.

***Tissue***

Distressed cells are placed at the center of the computational domain in a circular region of radius 175 $\mu$m. Cells are initialized at approximate equilibrium spacing with six neighbors (hexagonal packing). Fibroblasts and macrophages are placed randomly throughout the domain, excluding the 200 by 200 $\mu \text {m}$ square containing the distressed cells. Initial cell positions were held consistent across stochastic replicates. See Supplementary Material Table 10 for additional details.

***Comparison to previous modeling efforts***

Comparing to previous work in the field, Dallon et al. 2001 studied the impacts of TGF-$\beta$ on collagen deposition in wound healing (Dallon et al. 2001). In their model, fibroblasts deposit collagen (ECM density) in response to TGF-$\beta$. Comparing results between baseline TGF-$\beta$ and increased TGF-$\beta$, they find more collagen deposition resulting in slower agents and less penetration of cells into the wound. While caused by a different dynamic (concentration of a signaling molecule versus contact with a different cell type), this is comparable to the slowing of the fibroblasts as they increase ECM density followed by the inability of them to penetrate fully to the center of the wound. Note also that in this simple example, we are not modeling contact guidance whereas Dallon et al. do (Dallon et al. 2001).

### Basement Membrane Disruption Precipitating Transition from Local to Invasive Carcinoma

Here, we model the transition from a ductally-confined cancer to a locally invasive cancer, inspired by the transition of ductal carcinoma *in situ* (DCIS) to invasive carcinoma (Piersma et al. 2020). Cancer cell recruited fibroblasts degrade basement membrane (represented as high density ECM) enabling subsequent cellular invasion (Chang and Chaudhuri 2019; Strell et al. 2019; Winkler et al. 2020).

**Fig. 5:**
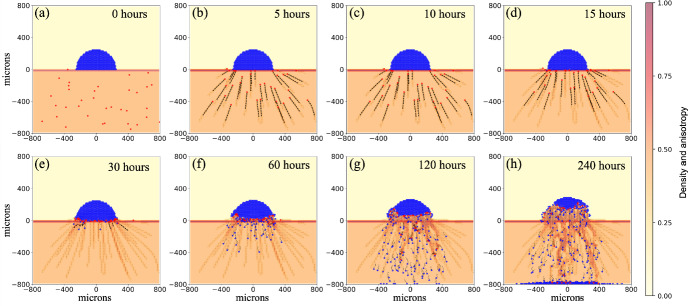
Dense ECM can form barriers while ECM degradation can lead to cell invasion: Beginning with a fluid-filled lumen, a layer of dense, impenetrable ECM, and stromal tissue **(a)**, we see that ECM modifying fibroblasts remodel the tissue locally **(b, c, d)**, enabling cell escape into surrounding tissue **(e, f, g, i)**. Initial configuration - cancer cells in half circle (175 $\mu \text {m}$ radius) with fibroblasts randomly placed outside duct. Red cells: fibroblasts. Blue cells: cancer cells. Yellow-to-red contour plot: ECM density and anisotropy (alpha = 0.5). Black arrows: cell positional history, going back eight time points (sampled every 30 minutes). This is available as a video. See Supplementary Material (Color figure online)

*Model results and cellular behavior details* In this model, there are two cell types - cancer cells and fibroblasts. The key rules of the model are summarized in Table 2. In this case, we simulate the duct’s longitudinal cross section. We start with an outgrowth of cancer cells (half circle of blue agents). The cells have grown into the fluid-filled region of low ECM density - the duct lumen. They are on a band of very dense ECM ($\rho$ = 1 - dark horizontal band in Fig. 5a) which represents the duct’s basement membrane. The basement membrane is surrounded by stroma - with ECM density of 0.5 (Fig. 5a). The cancer cells emit a tissue remodeling factor, modeled as an inflammatory factor, that attracts fibroblasts via chemotaxis with high bias (field not visualized). The fibroblasts alter the ECM and basement membrane, increasing anisotropy in the stromal region and degrading the basement membrane to a lower ECM density ($\rho _{\text {target}} = 0.5$) as they move into the tumor (Figs. 5b, c, and d). Due to close proximity of fibroblasts, cancer cells alter their adhesion and affinity to each other (Szabo et al. 2023), and escape, following the oxygen gradient originating at the lower simulation domain boundary (Figs. 5e, f). Because the cancer cells follow ECM, they preferentially move along the groomed ECM paths, or portions of high anisotropy, but also transit the ungroomed ECM in their movement out of their location of origin (Figs. 5g,i). See Supplementary Material Table 11 for additional details. A video of results presented in Figure 5 is available in the Supplementary Material.

**Table 2:** Invasive carcinoma model: principle behaviors, parameters, and systems effects. See Supplementary Material for parameter definitions

Cell type	Behavior	Key parameter(s)	Key effects
Cancer cells	Secrete inflammatory signal	Secretion rate, target value, and secretion rule parameters	Recruits fibroblasts
	ECM following	ECM sensitivity	Enables invasion of stroma
	ECM density response	$\rho _{\text {l}}$, $\rho _{\text {ideal}}$, $\rho _{\text {h}}$	Cell speed is reduced as ECM density moves away from ideal value (0.5)
	Chemotaxis up oxygen gradients	Bias	Enables escape
	Decrease in cell-cell adhesion on contact with fibroblast	Adhesion rule saturation and half-maximum values	Enables cells to more readily escape the tumor mass
	Decrease in cancer cell affinity on contact with fibroblast	Affinity rule saturation and half-maximum values	Enables cells to more readily escape the tumor mass
Fibroblasts	Chemotaxis up inflammatory gradients	Bias	Enables fibroblasts to find cancer cells
	ECM density response	$\rho _{\text {l}}$, $\rho _{\text {ideal}}$, $\rho _{\text {h}}$	Cell speed is reduced as ECM density moves away from ideal value (0.5)
	Degrading/depositing ECM density	ECM density target value	Removal of barriers to cell movement
	Altering ECM orientation and fiber-fiber alignment	ECM reorientation rate and anisotropy growth rate	Provides paths for cell escape
	Decrease speed on contact with cancer cells	Speed inhibition rule saturation value and half-maximum	Slows cell speed enabling remodeling to occur

All 21 stochastic replicates generated qualitatively similar results - basement membrane degradation and subsequent invasion of stroma. Figure 5 is a representative example. See the data supplement at Zenodo for individual results.

*Tissue microenvironment* We use two diffusive chemical fields in this simulation. Inflammatory signal is secreted by the cancer cells and has a diffusion length scale of 100 $\mu \text {m}$ in regions of high fibroblast density. We also simulate a nutrient field, modeled as oxygen (length scale of 1000 $\mu \text {m}$ in open tissue and 100 $\mu \text {m}$ in cell dense regions) coming into the duct from the bottom of the simulation. The inflammatory signal has no flux boundary conditions while the oxygen has mixed conditions: a constant value (Dirichlet condition) at the bottom of the domain and no flux conditions at the other boundaries.

The ECM begins with three zones. The top of the simulation, in which the cancer cells start, represents the interior portion of a duct (the lumen). We model it as completely fluid-filled with ECM density set to zero. The duct is surrounded by a region of very dense ECM which represents the basement membrane. Finally the duct itself is surrounded by stroma, which we model with an ECM density of 0.5. We are viewing the duct in a longitudinal cross section, rather than axially.

See the Supplementary Material Tables 12 and 13 for additional details.

*Tissue* Cancer cells are placed in the upper half of the domain in a half circular region of radius 175 $\mu$m with its main diameter in contact with the region of dense ECM (basement membrane). Cells are initialized at approximate equilibrium spacing with six neighbors (hexagonal packing). Fibroblasts are randomly placed throughout the lower portion of the domain, with placement held consistent across stochastic replicates. See the Supplementary Material Table 14 for additional details.

*Comparison to previous literature* Comparing to previous modeling results, Kim and Othmer investigated stromal invasion enabled by basement membrane degradation (Kim and Othmer 2013). In their model, also inspired by DCIS, the tumor cells themselves degrade the basement membrane by secreting proteolytic enzymes. In their model, as in ours, the breakdown of the ECM surrounding the mass of cells led to cellular invasion. In their case, they observed that a front of leading cells form as the basement membrane was broken down compared to our more dispersed breakdown of basement membrane and invasion. However, they had a point source of attractant compared to our line source. Furthermore, our basement membrane is broken in several locations and our tumor cells follow contact guidance. Both of these features tend to promote a spreading of the tumor cells compared to Kim and Othmer’s scenarios.

### ECM Contact Guidance Enables Leader-Follower Migration

To address questions centered on the mechanisms of multicellular invasion, we developed a tissue simulation focusing on collective cell migration. It is inspired by the cancer biology literature (Cheung et al. 2013; Carey et al. 2013) and builds off previous cell-based spheroid modeling work (Ghaffarizadeh et al. 2018). Mimicking Cheung et al. (2013), we include two cell phenotypes: more aggressive leader cells and less aggressive follower cells. Their observations suggest some form of communication between the leaders and followers; we model that communication as signals written in and read from ECM microstructure. We use our cell-ECM interaction model with leaders writing signals (remodeling microstructure) and followers reading those signals (contact guidance). Model details follow as well as more detailed accounting of the simulations that lead up to collective migration.

**Table 3:** Cell-based model: principle behaviors, parameters, and systems effects. See Supplementary Material for parameter definitions

Phenotype	Behavior	Key parameter(s)	Key effects
Leader cells	Altering ECM orientation and fiber-fiber alignment	ECM reorientation rate and anisotropy growth rate	Produces signals in ECM, providing paths for cell escape
	Chemotaxis	Bias	Enables production of coherent paths
	Cell-cell adhesion and repulsion	Ratio of adhesion strength to repulsion strength	Enables collective cell migration
Follower cells	ECM following	ECM sensitivity	Allows cells to read signals in the ECM
	Chemotaxis	Local anisotropy	Enables coherent migration
	Cell-cell adhesion and repulsion	Ratio of adhesion strength to repulsion strength	Enables collective cell migration
	Random motion	Local anisotropy	Enables finding of paths in the ECM

#### Leader-Follower Model Details

Cellular modeling We model two different phenotypes - leader cells and follower cells. In our example, these cells represent invasive and non-invasive cancer cells, respectively, but they could represent other types of cells that exhibit similar behaviors (more motile and less motile sub-phenotypes). The key rules of the leader-follower model are summarized in Table 3. In particular, leader cells follow oxygen gradients with a high chemotactic bias and encode their movements via changes to ECM microstructure, specifically altering ECM element orientation and anisotropy (see Supplementary Material Section 1 for mathematical details). Followers’ movements are influenced by signals in the ECM (contact guidance) and are chemotactic with bias equal to local value of *a* (Chemotaxis Model II - Supplementary Material Equation 16) following previous works that used chemotactic bias (McDougall et al. 2006; Martinson et al. 2023). They read signals in the ECM with a high sensitivity ($s = 1.0$) and are incapable of remodeling ECM. Thus, in highly anisotropic ECM, followers use contact guidance and chemotax, while in unaligned ECM, they move randomly. Combining leader and follower behavior together, leaders effectively signal paths which followers read through contact guidance. See Supplementary Material Table 15 for additional parameter values.

***Tissue microenvironment***

We simulate avascular tissue with a diffusing oxygen-like field coming from the domain boundaries (Dirichlet conditions). Cells uptake oxygen and there is decay in the cellular milieu. As in the basement membrane example, we use a diffusion length scale of 100 $\mu \text {m}$ for regions of high cell density. See Supplementary Material Table 16 for additional details.

The ECM is initialized uniformly with anisotropy of 0.0, ECM density of 0.5 (no impact on cell speed), and random fiber orientations. See Supplementary Material Table 17 for additional details.

***Tissue***

Cells are placed at the center of the computational domain in a circular region of radius 175 $\mu$m and were randomly assigned to be either followers or leaders (95% followers, 5% leaders). Cells are initialized at equilibrium spacing with six neighbors (hexagonal packing), with placement held constant across stochastic replicates. See the Supplementary Material Table 18 for additional details.

#### Leader-Follower Results

***Signal generation and reading are required for collective behavior***

**Fig. 6:**
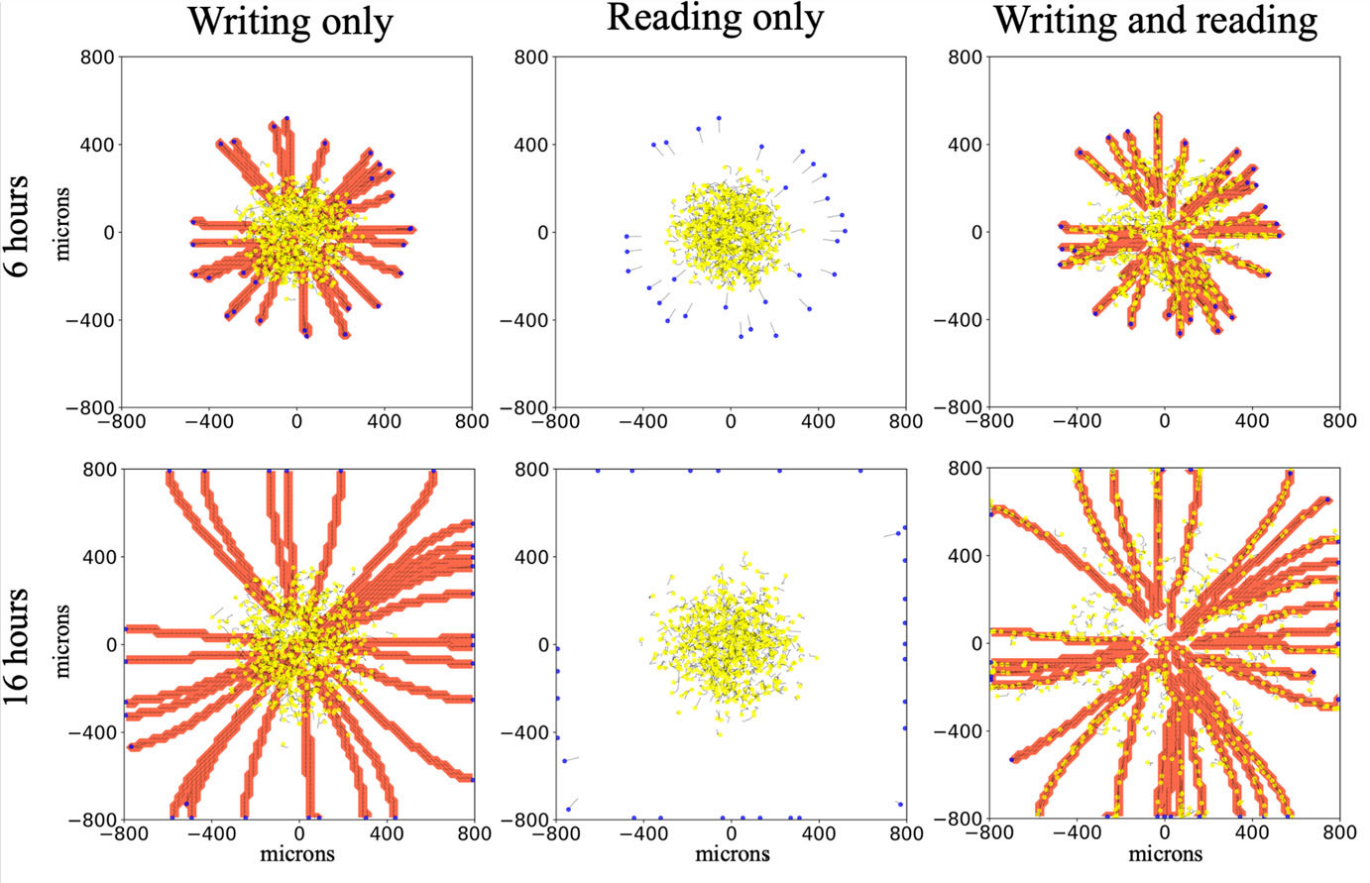
Communication of cell path through environmental modification produces stigmergic cell behavior: Modification of the environment (left) and reading of the environmental signals (middle) separately do not produce collective behavior. With both together (right column) we observe that most cells escape the domain center and invade their surroundings through the combined effects of following leaders’ paths (stigmergy) and chemotaxis. Writing only: *s*, ECM sensitivity, for followers set to zero. Reading only: $r_{\text {f}_{\text {0}}}$ and $r_{\text {a}_{\text {0}}}$, reorientation and realignment rates, for leaders set to zero. Initial configuration - 703 agents (5 % leaders) in 175 $\mu \text {m}$ circle. Yellow agents: followers. Blue agents: leaders. Red contour plot: ECM anisotropy - uniformly equal to one in remodeled ECM elements. Line segments: ECM orientation. Black arrows: cell positional history, going back 12 time points (sampled every 6 minutes). Videos are available. See Supplementary information (Color figure online)

We begin the exploration as a proof of concept with simplified dynamics: leader cells exhibit the limit or extreme behavior of instant ECM microstructure remodeling, using Supplementary Material Equations 1 and 2. (Note the previous examples, Sections 3.2 and 3.3, used continuous or non-instant remodeling - Supplementary Material Equations 3 and 5.) We use these simplified dynamics to aid exploration, not to suggest that cells necessarily modify ECM much faster than they move. Additionally, to show there is impact from the cell-ECM interactions, we isolate two model behaviors, the ability to write (matrix remodeling) and read signals (contact guidance), while disabling cell-cell adhesion. In Fig. 6, we observe similar results with ECM writing only (left column: follower ECM sensitivity *s* set to zero) and ECM reading only (middle column: leader remodeling rates set to zero). In both cases, leaders separate from the initial disc of cells and reach the simulation boundary at approximately 16 hours while followers remain roughly in the center of the domain. Only with both writing and reading (right column) were most cells able to escape the domain center and invade their surroundings. The guidance provided by the ECM combined with the chemotactic behavior of followers on groomed ECM enables them to follow the leaders’ paths out of the center of the domain. This results in stigmergy, wherein agents alter their environment and in doing so, effectively signal preferred paths to other agents. Videos are in the Supplementary Material.

Note that we hold the initial configuration of each simulation variation constant to isolate motility effects only.

***Cell-cell adhesion enables collective migration***

**Fig. 7:**
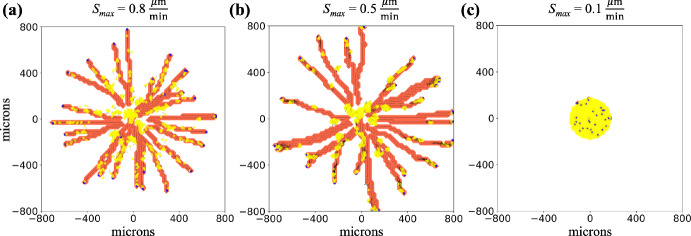
Multiple behaviors are produced with the leader-follower model by including cell-cell adhesion: With cell-cell adhesion on (adhesion = 10 $\frac{\mu \text {m}}{\textrm{min}}$) across all cell-cell interactions), multiple behavioral regimes are observed in the limit case of instant remodeling. In **(a)** with $S_{\textrm{max}}$ (maximum cell migration speed) at 0.8 $\frac{\mu \text {m}}{\textrm{min}}$, we see stigmergy with followers (yellow cells) following trails left by leaders (blue cells), as in Fig. 6. **(b)** Decreasing $S_{\textrm{max}}$ to 0.5 $\frac{\mu \text {m}}{\textrm{min}}$ yields collective migration. In **(c)** with $S_\textrm{max}$ at 0.1 $\frac{\mu \text {m}}{\textrm{min}}$, cell-cell adhesion cannot be overcome and the cell positions are frozen in the initial configuration. Initial configuration - 703 agents (5 % leaders) in 175 $\mu \text {m}$ circle. Adhesion set to 10 $\frac{\mu \text {m}}{\textrm{min}}$. Yellow agents: followers. Blue agents: leaders. Red contour plot: ECM anisotropy - uniformly equal to one in remodeled ECM elements. Line segments: ECM orientation. Black arrows: cell positional history, going back 12 time points (sampled every 6 minutes in **a**, every 30 minutes in **b**, and every 120 minutes in **c**). Videos are available. See Supplementary Material (Color figure online)

Adding cell-cell adhesion (see Supplementary Material Section 3 for details on the cell-cell adhesion model) to provide more realism to the simulation, while still using the limit assumption of instant signal writing, we observed a range of dynamics and cellular patterning, including collective migration in which phenotypically heterogeneous cells gather in clusters and move together while roughly maintaining the same composition over time. We see that ECM-mediated communication can still produce stigmergy (Fig. 7a) when speed is high enough to overcome cell-cell adhesion. In Fig. 7b, we see leaders migrated to the leading edge of cell clusters. Due to a balance between cell motility and cell-cell adhesion, cells stay in contact with each other, producing leader-follower collective invasion. Finally, when cell speed is even lower, adhesion takes over cell mechanics and relative cell positions are constant over the computational experiment (Fig. 7c) while there is a small overall displacement of the whole cell mass (see video). Simulation videos are in Supplementary Material. Note that cell-cell adhesion is of equal strength across all possible combinations of interactions (follower-follower, follower-leader, and leader-leader). Also, due to our interests in highlighting cell-ECM interactions, we have excluded exploring other possible ways to enable collective migration such as cell-cell repulsion with cell proliferation.

***Collective migration is retained in the context of non-instant signal generation***

**Fig. 8:**
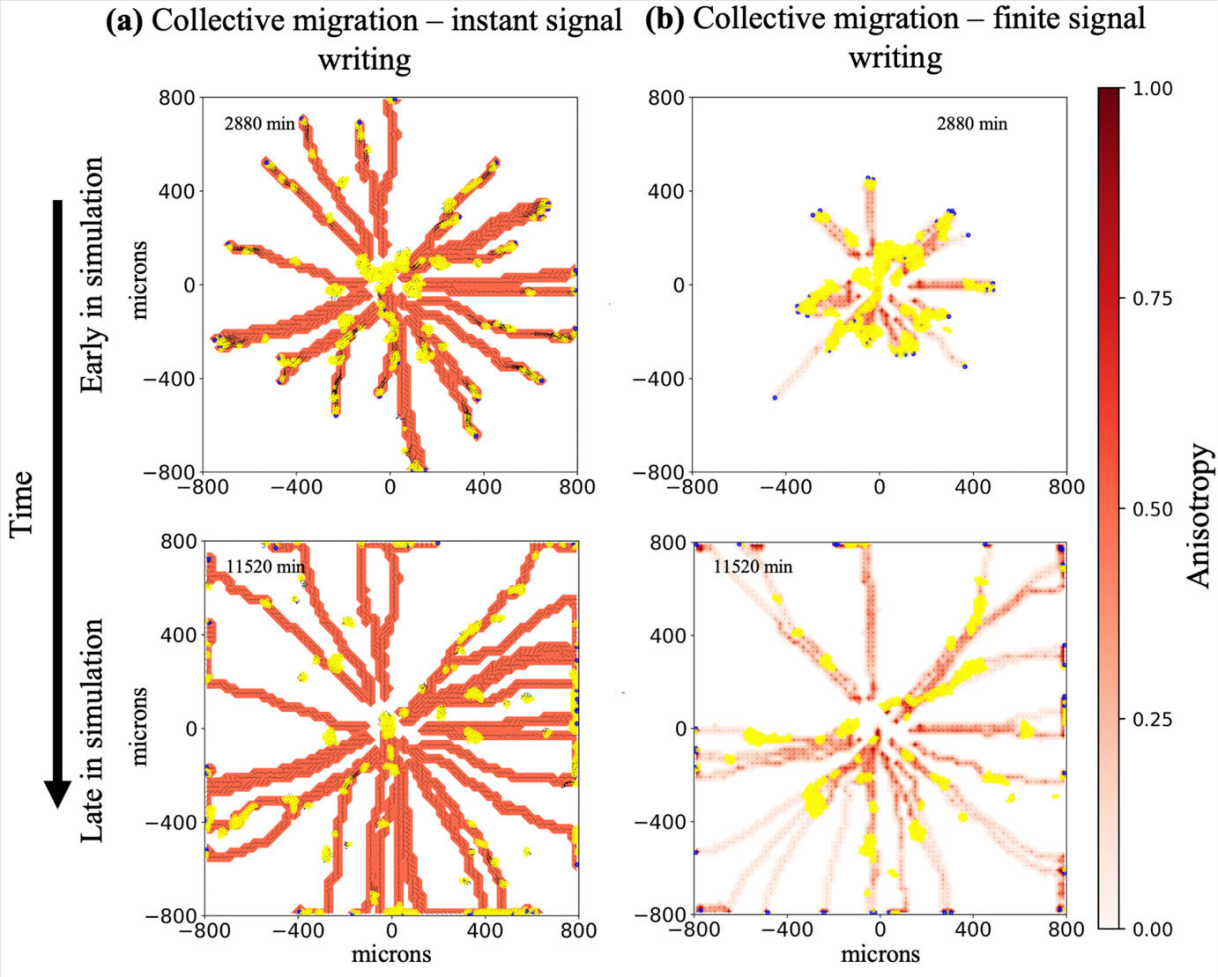
Collective migration is retained in the context of non-instant signal writing: In **(a)**, which is the limit case of instant signal generation (same parameter settings as Fig. **b**), we observe mixed clumps of both leader (blue) and follower (yellow) cells migrating together (leader-follower collective migration). In **b**, we relax instant remodeling and still observe leader-follower collective migration: leaders travel at the front of mixed clusters of leader and follower cells, leading them out of the center of the domain. They do not stay in contact as long as in **a** but strong enough paths are generated to enable followers to continue out via stigmergy. Fiber realignment rate: 0.004 $\hbox {min}^{-1}$, fiber reorientation rate: 4 $\hbox {min}^{-1}$. Initial configuration - 703 agents (5 % leaders) in 175 $\mu \text {m}$ circle. Adhesion set to 10 $\frac{\mu \text {m}}{\text {min}}$. Yellow agents: followers. Blue agents: leaders. Contour plots: ECM anisotropy - (**a**) uniformly equal to one in remodeled ECM elements and (**b**) color bar. Line segments: ECM orientation. Black arrows: cell positional history, going back 12 time points (sampled every 6 minutes). Videos are available. See Supplementary information (Color figure online)

Having shown that the cell-ECM model can produce a range of behaviors in the limit of instant signal generation, we now relax this assumption and let signal generation occur over a period of time instead of instantly. We give ECM reorientation and anisotropy non-instant rates of change and update them with Supplementary Material Equations 3 and 5, as in the previous example models. See Supplementary Material Section 1 for additional details. Figure 8 contrasts the results of the limit of instant signal writing with non-instant signal writing. Figure 8a shows two time points using the limit model, with cell speed of 0.5 $\mu \text {m}$/min and adhesion value of 10 $\mu \text {m}$/min (the same parameters as Fig. 7b). In the earlier time point (top), we see what was the initial cluster of cells broken into several smaller clusters moving out from the center of the domain. In the next time point, we see that heterogenous clusters have continued towards the simulation boundary (with some reaching it) while clusters of followers remain in the middle. In Fig. 8b, relaxing instantaneous signal writing, we observe that collective migration still occurs. Leaders alter ECM with the amount of microstructure change, and thus signal strength, depending on cellular residence time, speed, and density, leading to a range of anisotropy values. Leaders remain in contact with followers and lead them out of the center of the domain. However, because altering ECM microstructure (in particular increasing anisotropy) is no longer instant, the signals to followers are not as strong compared to 8a. We observe that fewer cells are at the periphery of the domain when remodeling is slower (finite). The relative lack of strong signals in the non-instant scenario leads to less chemotactically biased migration (more random migration) in the follower population, decreasing the uniformity of velocities across a cluster of cells. Thus, as the cells adhere to each other but attempt to move in divergent directions, overall cell speed is effectively reduced (see Supplementary Material Equation 21). The more uniform the cell velocities, the closer to $S_{\text {max}}$ the average speed of a cluster will be. Overall, the lack of uniformity leads to less coherent migration in which the consistently chemotactically-driven leaders break away and are then able to proceed ahead of the followers. For more details see Supplementary Material Section 3 and Equation 19. The remaining followers then continue out behind the leaders via stigmergic behavior. Videos are available in the Supplementary Material.

We performed stochastic replicates on the continuous remodeling collective migration simulation of which Fig. 8b is a representative example. All 21 stochastic replicates generated similar results - leader-follower type invasion followed by continued stigmergic movement of followers along the leader generated ECM paths. See the data repository at Zenodo for individual results.

We observed that collective migration behavior in the finite signal writing scenario is sensitive to remodeling parameters and is lost with a less than order of magnitude change in fiber realignment and reorientation rates as seen in Supplementary Material Figure 5. Fiber reorientation and realignment rates varying by a factor of four are enough to change the behavior. This model could be further tuned, matching to data related to cell speeds and patterning, to suggest conditions under which collective leader-follower migration may occur in non-computational model systems.

Examining Figs. 6 and 7, in Fig. 6 (without cell-cell adhesion), follower cells engage in stigmergy (right panel) while frequently straying from leader-cell created paths. In contrast, in Fig. 7a (with cell-cell adhesion), straying is less frequent, implying that cell-cell adhesion enhances stigmergy, an observation deserving further study.

This model could also explore “wisdom-of-the-crowd" effects in noisily oriented ECM. Prior work has shown that the cell clusters average out noise in chemotactic gradients better than single cells (Colizzi et al. 2020). Additionally, cell-ECM sensitivity, ECM anisotropy, and cell-cell adhesion could be varied to study impacts of other aspects of cell-ECM and cell-cell interactions on these effects.

Finally, we note that in this example model, we did not explore asymmetric rates of cell-cell attachment. Spring-like attachments with different rates of attachment versus detachment may better mimic cell-cell adhesion (Ozik et al. 2019). This enhanced adhesion might enable leaders to better pull cells and produce collective migration, perhaps eliminating the need for the followers’ oxygen-driven directional cue.

***Comparison to other works***

Comparing to previous biological and modeling work in the field, Ilina et al. studied collective migration and cancer metastasis, attempting to understand connections between cell-cell adhesion, cell density, and ECM confinement (Ilina et al. 2020). Their *in vitro* and *in vivo* experimental results showed that collective migration occurs with high cell-cell adhesion but also with low cell-cell adhesion (for example with knocked down E-cadherin expression) in the context of high ECM density, which corresponds to relatively decreased speed. Furthermore, the phenomena of collective behavior was investigated with an agent-based model of cells transiting along a collagen ECM and plastic culture dish interface. In particular, the authors found that low cell-cell adhesion and low ECM density, which led to higher cell speed, were associated with individual cell migration, while high ECM density (lower cell speed due to ECM confinement) or sufficiently high cell-cell adhesion both enabled collective migration. The high ECM density/low cell-cell adhesion lacked local cell to cell velocity correlations compared to the low ECM density/high cell-cell adhesion. They suggest that the system’s jamming-unjamming phase transitions, which describe the shift between a solid-like state (cells are jammed and largely immobile) and a fluid-like state (cells are unjammed and can move individually or collectively), may have several phases. These include a jammed phase, two mobile phases corresponding to the two collective migration regimes (one with low cell-cell adhesion and another with high cell-cell adhesion), and finally an unjammed gas-like phase. While we did not look at ECM-based cell confinement, this bears similarity to our results in Fig. 7, with the gas phase corresponding to 7a (ECM of sufficiently low density to not confine cells), 7b corresponding to the active nematic regime, and 7c being jammed, but in this case strictly due to cell-cell adhesion. Following up on Ilina et al., Kang et al. studied collective cell migration in tumor spheroids with a hybrid vertex-particle-based model, culminating in a phase diagram of cell jamming (Kang et al. 2021). They found that in the regime of low cell speed and higher ECM density (more regions of excluded volume within the domain), cells remained clumped together (solid phase) until a sufficient speed was reached to enable collective movement (liquid phase). Finally, with lower ECM density (relatively less volume exclusion for cells) and higher cell speeds a gas-like phase was observed. Again, these results are similar to our results in Fig. 7 with the gas phase corresponding to Fig. 7a, liquid phase to 7b and solid to 7c. Interestingly, both Ilina et al. (2020) and Kang et al. (2021) either hint at or produce phase diagrams. Future work with this example model could explore the transitions between the subplots of Fig. 7 as a phase diagram, in addition to considering aspects of cell confinement through use of a non-uniform initialization of the ECM and the possible role of heterogenous local chemotactic gradients in the formation of ECM paths.

Finally, while the collective migration example was inspired by the cancer biology literature (Cheung et al. 2013; Carey et al. 2013), Martinson et al. (2023) produced a similar model. In this work, they model neural crest cell invasion, also using ECM remodeling cells to lead clusters of ECM following cells into tissue. While varying in some aspects (such as using haptotaxis and only cell-cell repulsion), their use of a similar leader-follower model in developmental biology hints at this model’s and the framework’s applicability to more general leader-follower migration models.

## Discussion

In this work, we presented a framework designed to capture salient aspects of bidirectional local cell-ECM interactions using a relatively simple mathematical model. Drawing on previous works, we developed a model of extracellular matrix using a mesh of voxels (ECM elements), each described by a limited set of features: overall anisotropy, overall orientation, and overall density. We added cell-ECM interactions including ECM-mediated changes in cellular motility (tunable contact guidance and ECM dependent cell migration speed) and cell-mediated local microstructure remodeling (changes to ECM element anisotropy, overall fiber orientation and density). Furthermore, through the use of PhysiCell rules (Johnson et al. 2023), additional bidirectional interactions are possible without the need to write, compile, and test custom interaction code. We demonstrated the framework with four examples that focus on cell motility-ECM interactions: an invasive cellular front, wound healing, basement membrane degradation, and collective migration. Each of these examples uses the same core framework with changes in source code only for simulation initialization. We make this newly implemented mesoscale, bidirectional cell-ECM interaction framework freely accessible to the community aiming to encourage and enable sharing of expertise among multiple problem domains (e.g., tumor biology, developmental biology, wound healing, angiogenesis, fibrosis). Furthermore, our approach opens up rich explorations of cell-matrix biology by allowing direct links between a broad array of cell behaviors and ECM variables, without requiring additional coding. This represents a key methodological advance in this work: enabling the exploration of a large set of relationships between cell behaviors and ECM state.

With this base framework constructed, there are opportunities for extensions. Future models could initialize the ECM variables from high-resolution ECM data based upon analysis of the mean and variation in fiber angles (for orientation and anisotropy) and amount of material present (for fiber density). We note that our framework currently captures local interactions between single ECM elements and single cells; it does not incorporate larger-scale interactions. This contrasts with other frameworks and models that represent the network like connections within a spatially-coupled ECM or distribute cellular remodeling and contact guidance among multiple ECM elements (e.g. - Zhu and Mogilner 2016; van Oers et al. 2014; Dallon et al. 1999). To address non-local remodeling of ECM, future efforts will focus on incorporating the option for a continuous, force-based ECM model, for example as a visco-poroelastic material using a finite element approach, then sampling the variables established in this work on the ECM element grid. To address the current limit of remodeling only a single ECM element, we aim to explore approaches to spread remodeling over multiple, adjacent ECM elements, for example with Gaussian smoothing or other techniques as well as enabling ECM influence on cell behavior from multiple ECM elements (Martinson et al. 2023; Dallon et al. 1999). Similarly, we aim to extend bidirectional cell-ECM interactions to allow cells spanning multiple elements to influence and be influenced by each element.

As noted in Section 2.2.2, due to ECM discretization, cells may miss ECM cues in regions of rapidly changing ECM orientation (high amounts of path curvature) as observed in Fig. 2b. ECM following is a function of cell speed, curvature in an ECM path, and element size. To reduce the effect of the discretization, as needed, ECM element size can be decreased by modelers noting that decreasing element size will be balanced by decreasing the area of influence in the bidirectional cell-ECM interaction and will increase computational costs. The current default size of 20 $\mu \text {m}$ per side was selected to be on the order of the size of the cell, as in a previous work (Ghaffarizadeh et al. 2018). This enables cells to modify a volume about equal to their volume, forming paths about one cell width in diameter (with our cell diameter of $\approx$ 17 $\mu \text {m}$). Future versions of the framework that include spreading the bidirectional cell-ECM influence over a region (discussed above) might ameliorate discretization effects by effectively smoothing the path.

In the initial version of the framework, we aimed to have simple constitutive relationships that produced a range of emergent behaviors showing the utility of the underlying framework variables and relationships. As an example, we model the anisotropy as only stable or increasing. This limits the ability to capture multiple, extensive remodelings of an ECM element. Future extensions to the framework could model both growth and decline in fiber-fiber alignment. Another possible extension is stochastic contact guidance where cell direction is sampled from a distribution rather than set deterministically. This approach was recently implemented using the von Mises distribution (Martinson et al. 2023), showing its feasibility and interest within the community. Moreover, in future releases, we plan to generalize the framework architecture to enable modelers to easily replace built in constitutive relationships with their own relationships. Furthermore, architecture that allows easy exchange of one constitutive relationship for another would enable useful comparisons among model relationships. Future work could include comparing simulation outcomes, numerics, and computational performance of cell-ECM interaction formulations as similarly investigated for cell-cell mechanics constitutive relationships (Mathias et al. 2020).

Finally, we note that the sample models were developed as 2-D simulations (single slice of a 3-D environment), and could be, by reasonable extension of the cell-ECM interaction models to 3-D, be extended to 3-D. Also, while we have demonstrated this framework for a generalized ECM or single ECM component, the framework could readily be expanded to support a vector of ECM components. These extensions are additional future work for the framework.

## Supplementary Information

Below is the link to the electronic supplementary material.

Supplementary file 1 (pdf 1853 KB)

Supplementary file 2 (mp4 3729 KB)

Supplementary file 3 (mp4 1306 KB)

Supplementary file 4 (mp4 672 KB)

Supplementary file 5 (mp4 12035 KB)

Supplementary file 6 (mp4 10003 KB)

Supplementary file 7 (mp4 13989 KB)

Supplementary file 8 (mp4 11750 KB)

Supplementary file 9 (mp4 2763 KB)

Supplementary file 10 (mp4 3700 KB)

Supplementary file 11 (mp4 9040 KB)

Supplementary file 12 (mp4 7708 KB)

Supplementary file 13 (mp4 9063 KB)

Supplementary file 14 (mp4 25100 KB)

Supplementary file 15 (mp4 9992 KB)

Supplementary file 16 (mp4 339 KB)

Supplementary file 17 (mp4 15474 KB)

Supplementary file 18 (mp4 388 KB)

Supplementary file 19 (mp4 11762 KB)
