# Supplementary material for: A Simple Framework for Agent-Based Modeling with Extracellular Matrix
Source: Bull Math Biol. 2025 Feb 12;87(3):43. doi: 10.1007/s11538-024-01408-8 (PMC11821717; doi:10.1007/s11538-024-01408-8)
Supplement: Supplementary file 1 — (pdf 1853 KB) [file 11538_2024_1408_MOESM1_ESM.pdf]

Supplementary Material for Metzcar et al. *A simple framework for agent-based modeling with extracellular matrix*

1 Cell remodeling of ECM microstructure

Here we detail the mathematical models for updating ECM element variables anisotropy, orientation, and density. The ECM elements and cells are conceived of as being in close contact; cells alter the ECM element they are physically in. To formalize which ECM element a cell modifies out of the many possible options (see Figure 1a of the main text), we define cells as interacting with whichever element their center coordinates are the closest to, as in [1]. To do this, suppose cell i is at position $\mathbf{x}_i$. We remodel the ECM element whose center $\mathbf{x}_i^*$ is closest to $\mathbf{x}_i$.

We start with outlining two related methods for altering ECM microstructure - the limit case of instantaneous remodeling and the non-instantaneous case.

Instant anisotropy and orientation remodeling

To explore an extreme case of ECM microstructure modification/ECM mediated communication, we model anisotropy and ECM element orientation as being immediately updated when contacted by a remodeling cell, resulting in the following relationships to update a and $\mathbf{f}$:

$$a(\mathbf{x}_i^*) = 1 \tag{1}$$

$$\mathbf{f}(\mathbf{x}_i^*) = \mathbf{d}_{\text{remodeler}}. \tag{2}$$

Because of the underlying updates to $\mathbf{f}$ (see next subsection), this permanently writes with high signal strength the motility direction of the first cell to pass through a particular ECM element.

Non-instantaneous microstructure remodeling

Anisotropy

Anisotropy is represented by a scalar ranging from 0 to 1, representing "complete lack of anisotropy" (or a disordered state) to "complete anisotropy" (a highly ordered, oriented state). The rate of change in anisotropy over time is given as:

$$\frac{da(\mathbf{x}_i^*)}{dt} = r_{\text{a}}(1 - a(\mathbf{x}_i^*)). \tag{3}$$

r_{a} is specified as:

$$r_{\text{a}} = r_{\text{a}_0} s_{\text{cell}} \tag{4}$$

where r_{a_0} is a base rate of change and s_{cell} is the migration speed of the cell changing the ECM element's anisotropy.

Orientation

We model changes to fiber orientation as follows:

$$\frac{d\mathbf{f}(\mathbf{x}_i^*)}{dt} = -r_\mathrm{f}\left(\mathbf{f}(\mathbf{x}_i^*) - \frac{\mathbf{d}_\mathrm{remodeler}}{||\mathbf{d}_\mathrm{remodeler}||}\right) \tag{5}$$

with

$$r_\mathrm{f} = r_{\mathrm{f}_0} s_\mathrm{cell}(1-a). \tag{6}$$

r_{f_0} is the base rate of change, $\mathbf{d}_\mathrm{remodeler}$ is the direction of the remodeling cell, and s_cell is cell speed. $\mathbf{f}$ is normalized after each update. Note that when $a = 1$, orientation cannot change. Also, we select to remodel $\mathbf{f}$ such that it takes on the smallest angle between it and $\mathbf{d}_\mathrm{remodeler}$.

Density

Density ranges from 0 to 1, representing a region lacking ECM material to a very dense region completely filled with ECM material, respectively. Cells change ECM density locally through the following relationship:

$$\frac{d\rho(\mathbf{x}_i^*)}{dt} = r_\mathrm{density}(\rho_\mathrm{target} - \rho(\mathbf{x}_i^*)) \tag{7}$$

where r_density is the cell's characteristic rate of deposition/degradation of ECM and ρ_target is the cell's target for ECM density. This parallels chemical substrate secretion by cells in PhysiCell [1]

2 ECM microstructure influence on cell motility

Cell motion, in particular, cell speed and direction, can be altered via cell-ECM interactions. ECM element orientation and anisotropy are combined with a cell's preferred direction to produce the cell motility vector. Independently, cell speed is influenced by ECM density enabling ECM to block or guide cell paths. We lay out the orientation and anisotropy influences first, then the influence of density. As with remodeling ECM microstructure, we define cells to be affected by and read whichever element their center coordinates are nearest as in [1].

2.1 ECM orientation and anisotropy

We developed a method to calculate $\mathbf{d}_\mathrm{actual}$, a cell's actual motility direction, based on the cell's preferred direction $\mathbf{d}_\mathrm{preferred}$, a cell specific ECM sensitivity s, and local anisotropy and orientation. As illustrated in SM Figure 1, we want to constrain cell motion to the sector spanning the ECM orientation ($\mathbf{f}$) and the cell's intended direction of travel ($\mathbf{d}_\mathrm{preferred}$) in a way that does not bias direction of travel along the fibers. We use $\mathbf{f}$ and $\mathbf{d}_\mathrm{preferred}$ to form an orthonormal basis set that spans the cell's potential directions (the sector produced by the angle θ in SM Figure 1. We calculate $\mathbf{d}_\mathrm{actual}$ by blending

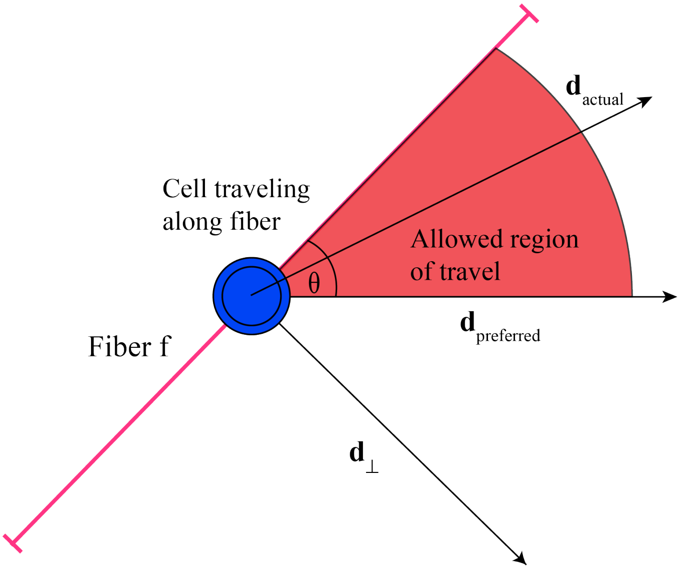

Supplementary Figure 1: Schematic for mathematical model of cell motility and ECM orientation interactions. Cell shown in blue. See 8, 10, and 12 for variable explanations.

the basis vectors using the cell's ECM sensitivity parameter s and local ECM element's anisotropy a as follows:

$$\mathbf{d}_{\text{actual}} = (1-\gamma)C_1\mathbf{d}_{\perp} + C_2\mathbf{f}, \tag{8}$$

where γ is the effective ECM influence parameter. This captures both cell sensitivity and magnitude of ECM fiber alignment (signal strength):

$$\gamma = a(\mathbf{x_i}^*) \cdot s_i. \tag{9}$$

s_i and $a(\mathbf{x}_i^*)$ are the sensitivity of cell i and anisotropy at that location respectively. The other terms in SM Equation 8, the coefficients C_1 and C_2 and basis vector $\mathbf{d}_{\perp}$, are calculated by decomposing $\mathbf{d}_{\text{preferred}}$ and basic trigonometry. For brevity, we will drop location and indexing of cells unless needed for clarity.

We find $\mathbf{d}_{\perp}$, using the trigonometric relationship $\mathbf{A} \cdot \mathbf{B} = ||\mathbf{A}||||\mathbf{B}||\cos\,\theta$. Letting $\mathbf{f}$ and $\mathbf{d}_{\text{preferred}}$ be unit vectors and θ being the angle between them, the relationship simplifies to:

$$\mathbf{d}_{\text{preferred}} \cdot \mathbf{f} = \cos\,\theta. \tag{10}$$

Scaling $\mathbf{f}$ by that dot product, we get the component of $\mathbf{d}_{\text{preferred}}$ that points along $\mathbf{f}$. Subtracting that result from $\mathbf{d}_{\text{preferred}}$, we obtain a vector perpendicular to $\mathbf{f}$ as follows:

$$\mathbf{d}_{\perp}^{'} = \mathbf{d}_{\text{preferred}} - (\mathbf{d}_{\text{preferred}} \cdot \mathbf{f})\mathbf{f} \tag{11}$$

with normalization producing the unit vector $\mathbf{d}_{\perp}$ seen in SM Equation 8 and SM Figure 1.

Note also that $\mathbf{d}_{\text{preferred}}$ can be decomposed as follows:

$$\mathbf{d}_{\text{preferred}} = C_1\mathbf{d}_{\perp} + C_2\mathbf{f} \tag{12}$$

with C_1 and C_2 the same as in SM Equation 8. They are determined via dot product and again, we take advantage of the terms being unit vectors:

$$\begin{aligned}\mathbf{d}_{\text{preferred}} \cdot \mathbf{d}_{\perp} &= (C_1\mathbf{d}_{\perp} + C_2\mathbf{f}) \cdot \mathbf{d}_{\perp} \\ &= C_1\mathbf{d}_{\perp} \cdot \mathbf{d}_{\perp} + C_2\mathbf{f} \cdot \mathbf{d}_{\perp} \\ &= C_1. \end{aligned} \tag{13}$$

Likewise, dotting both sides of SM Equation 12 by $\mathbf{f}$, we obtain C_2 as follows:

$$\begin{aligned}\mathbf{d}_{\text{preferred}} \cdot \mathbf{f} &= (C_1\mathbf{d}_{\perp} + C_2\mathbf{f}) \cdot \mathbf{f} \\ &= C_1\mathbf{d}_{\perp} \cdot \mathbf{f} + C_2\mathbf{f} \cdot \mathbf{f} \\ &= C_2. \end{aligned} \tag{14}$$

With that, all required parts of SM Equation 8 are determined, letting $\mathbf{d}_{\text{actual}}$ be calculated. Note that if the effective influence parameter γ is zero, SM Equation 8 becomes SM Equation 12, meaning $\mathbf{d}_{\text{actual}}$ will equal $\mathbf{d}_{\text{preferred}}$. Likewise, when γ is one, its maximum value, $\mathbf{d}_{\text{actual}}$ equals $\mathbf{f}$.

In the case that $\mathbf{d}_{\text{preferred}}$ is parallel to $\mathbf{f}$, $\mathbf{d}_{\perp}^{'}$ from SM Equation 11 is the zero vector. We assume the norm of the zero vector to be zero. With that set, the calculations will proceed as normal.

Note that there are two possibilities on how to interpret $\mathbf{d}_{\text{actual}}$:

A: The cell senses its environment, determines direction of travel along the fibers, and all cell effort goes into that motion.

B: The cell attempts to move both along the fibers and along its preferred direction, wasting effort in the process.

These two interpretations lead to either normalizing $\mathbf{d}_{\text{actual}}$ (Option **A**) or letting the cell direction vector lose magnitude by not normalizing $\mathbf{d}_{\text{actual}}$ (Option **B**). For the purposes of this work, we choose Option **B** and do not normalize $\mathbf{d}_{\text{actual}}$.

Constitutive relations to determine $\mathbf{d}_{\text{preferred}}$

To determine $\mathbf{d}_{\text{preferred}}$ in SM Equation 8, we use either the motility model of [1]'s motility model or an adaptation that directly incorporates anisotropy.

Model I: Chemotaxis based motility

To determine $\mathbf{d}_{\text{preferred}}$ for chemotactically motivated cells, we use the following equation from [1]:

$$\mathbf{d}_{\text{preferred}} = (1 - b) \cdot \mathbf{d}_{\text{random}} + b \cdot \mathbf{d}_{\text{chemotaxis}} \tag{15}$$

where $\mathbf{d}_{\text{random}}$ is a randomly generated unit vector, $\mathbf{d}_{\text{chemotaxis}}$ is a unit length microenvironment-derived direction, such as the gradient of a cell-required substrate, and the scalar b, running from 0 - 1, is the directional bias parameter that sets the balance between random (b=0) and completely biased cell motility

r, (b=1). $\mathbf{d}_{\text{preferred}}$ is the cell-selected motility direction and may be combined with local fiber orientation to produce the final cell motility direction.

Model II: ECM and chemotaxis based motility

To couple chemotactic behavior with reading of ECM signals, we reformulated Equation 15 and replace the chemotactic bias with the local ECM anisotropy:

$$\mathbf{d}_{\text{preferred}} = (1 - a) \cdot \mathbf{d}_{\text{random}} + a \cdot \mathbf{d}_{\text{chemotaxis}} \tag{16}$$

Here, a replaces the bias parameter in setting the random versus chemotactic behavior. When combined with the cell-ECM interactions, this model effectively enables a cell to only be chemotactic when in an element of anisotropic ECM.

In either model, $\mathbf{d}_{\text{preferred}}$ is updated stochastically, with an average update interval equal to the cell's persistence time.

2.2 ECM density

We use the following relationship to incorporate the influence of ECM density ρ on cell speed:

$$s_{\text{cell}} = \begin{cases} 0 & \text{if } \rho \leq \rho_{\text{l}} \\ S_{\text{max}} \frac{\rho - \rho_l}{\rho_{\text{ideal}} - \rho_{\text{l}}} & \text{if } \rho_{\text{l}} < \rho \leq \rho_{\text{ideal}} \\ S_{\text{max}} \frac{\rho - \rho_h}{\rho_{\text{ideal}} - \rho_{\text{h}}} & \text{if } \rho_{\text{ideal}} < \rho < \rho_{\text{h}} \\ 0 & \text{if } \rho \geq \rho_{\text{h}} \end{cases} \tag{17}$$

where ρ is the local ECM density, S_{max} is the maximum cell migration speed, ρ_{l} is the ECM threshold density above which cells have non-zero speed, ρ_{h} is the ECM threshold density above which cells cannot move, and ρ_{ideal} is the ECM density of maximum speed. Supplementary Figure 2 shows the tent-like shape of SM Equation 17 with the parameters used in our example models.

This form simulates a cell that in a fibrous environment cannot move either when density is too low, due to a lack of fibers to grab onto for propulsion, or when density is too high, meaning the ECM is too dense for the cell to pass through. Other polynomials or curves derived from fitting to data could be used in place of this form. We choose it to be a minimum example of a tunable, potentially asymmetric curve with a local maximum.

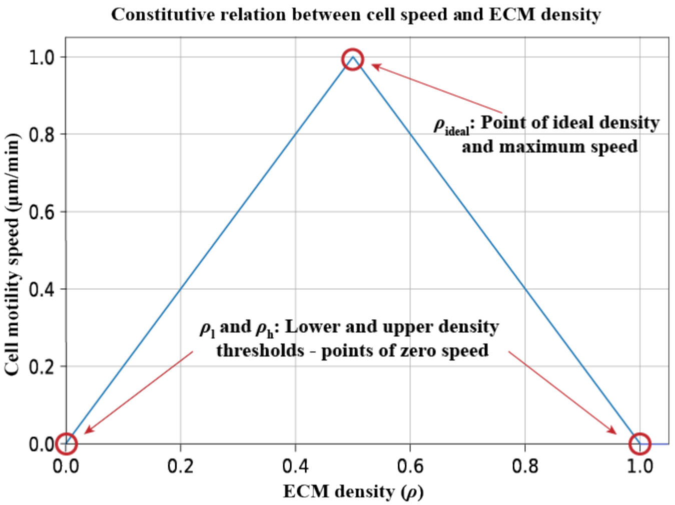

Supplementary Figure 2: Constitutive relationship between cell speed and density. This is the graphical form of SM Equation 17 with parameters used for the example models.

2.3 Combined ECM microstructure and chemical environment influence on cell motility

Combining all these influences together produces the equation for cell velocity due to cell motility (locomotion):

$$\mathbf{v}_{\text{loc}} = s_{\text{cell}} \mathbf{d}_{\text{actual}} \tag{18}$$

where s_{cell} is from SM Equation 17 and $\mathbf{d}_{\text{actual}}$ is from SM Equation 8.

3 Cell velocity and cell-cell adhesion and repulsion model

In PhysiCell, the agent-based modeling framework in which our examples and ECM model are built, cell velocities and positions update based on several forces - cell-cell forces as well as cell-environmental forces and cell-locomotive force. In this current work, we focused on cell-cell forces (cell-adhesion and repulsion) as well as cell locomotive forces. To discuss this, we follow the explanation from [1], beginning with the cell equation of motion. See [1] and [2] for additional details and discussion.

Given a cell i at position $\mathbf{x}_i(t)$, velocity $\mathbf{v}_i(t)$ and a set of neighboring cells $\mathcal{N}(i)$, PhysiCell models cell motion with this equation:

$$m_i \dot{\mathbf{v}}_i = \sum_{j \in \mathcal{N}} (\mathbf{F}^{ij}_{\mathrm{cca}} + \mathbf{F}^{ij}_{\mathrm{ccr}}) + \mathbf{F}^{i}_{\mathrm{drag}} + \mathbf{F}^{i}_{\mathrm{loc}} \tag{19}$$

where $\mathbf{F}_{\mathrm{cca}}$ and $\mathbf{F}_{\mathrm{ccr}}$ are the cell-cell adhesive and repulsive (cell resistance to deformation) forces, $\mathbf{F}_{\mathrm{drag}}$ represents dissipative, drag-like forces such as viscous drag, and $\mathbf{F}_{\mathrm{loc}}$ is cell generated, or motile, force. Note that as appropriate, additional force terms can be added (see [1] and [2]).

Examining this term by term, drag is modeled as follows [1–3]:

$$\mathbf{F}_{\mathrm{drag}} = \nu \mathbf{v}_i. \tag{20}$$

Here, ν is a drag coefficient. This differs from the formulation in [1], which also included a drag term for ECM interactions. Since we model ECM interactions explicitly in the update for cell locomotive force, we do not it include it here. The formulation makes the inertialess assumption ($m_i \dot{\mathbf{v}}_i \approx 0$) [1, 2, 4, 5] which assumes that any changes to forces on the cell rapidly equilibrate. Making these substitutions in Equation 19 and solving for $\mathbf{v}_i$, we get:

$$\mathbf{v}_i = \frac{1}{\nu} \left(\sum_{j \in \mathcal{N}} (\mathbf{F}^{ij}_{\mathrm{cca}} + \mathbf{F}^{ij}_{\mathrm{ccr}}) + \mathbf{F}^{i}_{\mathrm{loc}} \right). \tag{21}$$

As noted in [2], this assumption yields the interpretation of each term as the terminal velocity of the cell, given that only the force of interest and drag are acting on the cell. The locomotive force and its contribution to cell velocity is covered in the previous section (2). The forces $\mathbf{F}_{\mathrm{cca}}$ and $\mathbf{F}_{\mathrm{ccr}}$ are then modeled using interaction potentials that are functions of maximum adhesion distance, cell geometry (radius), adhesion and repulsion parameters, and distance to other cells. The adhesion function is:

$$\mathbf{F}^{ij}_{\mathrm{cca}} = -C^{*}_{\mathrm{cca}} A_i A_j \nabla \phi_{n_{\mathrm{cca}}, R_{i,\mathrm{A}} + R_{j,\mathrm{A}}} (\mathbf{x}_j - \mathbf{x}_i), \tag{22}$$

where C^{*}_{cca} is the cell-cell adhesion parameter, A_i and A_j are the cell-cell relative adhesion parameters (bounded inclusively between 0 and 1), ϕ is the potential function, n_{cca} a parameter setting the shape of the potential function, and $R_{i,\mathrm{A}}$ and $R_{j,\mathrm{A}}$ are the maximum adhesion distances of cells i and j respectively. Note that this gives C^{*}_{cca} units of force. To define the mechanics parameter in PhysiCell, we divide through by ν giving:

$$C_{\mathrm{cca}} = \frac{1}{\nu} C^{*}_{\mathrm{cca}} \tag{23}$$

with C_{cca} taking on units of speed, as expected for SM Equation 21.

This is the repulsion function:

$$\mathbf{F}^{ij}_{\mathrm{ccr}} = -C^*_{\mathrm{ccr}} \nabla \psi_{n_{\mathrm{ccr}}, R_i + R_j}(\mathbf{x}_j - \mathbf{x}_i), \tag{24}$$

where C^*_{ccr} is the cell-cell repulsion parameter, n_{ccr} is a parameter setting the shape of the potential function, and R_i and R_j are the cell radii of the two interacting cells i and j. As above with C^*_{cca}, we divide C^*_{ccr} by ν:

$$C_{\mathrm{ccr}} = \frac{1}{\nu} C^*_{\mathrm{ccr}} \tag{25}$$

giving C_{ccr} units of speed.

Finally, PhysiCell uses the following potential functions for adhesion and repulsion respectively:

$$\nabla \phi_{n,R_{\mathrm{A}}}(\mathbf{r}) = \begin{cases} \left(1 - \frac{|\mathbf{r}|}{R_{\mathrm{A}}}\right)^{n+1} \frac{\mathbf{r}}{|\mathbf{r}|} & \text{if } |\mathbf{r}| \leq R_{\mathrm{A}} \\ \mathbf{0} & \text{otherwise,} \end{cases} \tag{26}$$

and:

$$\nabla \psi_{n,R}(\mathbf{r}) = \begin{cases} -\left(1 - \frac{|\mathbf{r}|}{R}\right)^{n+1} \frac{\mathbf{r}}{|\mathbf{r}|} & \text{if } |\mathbf{r}| \leq R \\ \mathbf{0} & \text{otherwise.} \end{cases} \tag{27}$$

Note that to compute the velocity updates, only the gradients of the potentials are required. $\mathbf{r}$ is the vector displacement between the interacting cells' centers, R_{A} is the sum of i and j's interaction distances, and R is the sum of the two cells' diameters.

4 PhysiCell rules

PhysiCell rules is a new extension to PhysiCell [6]. It uses a grammar to encode a mathematical representation of cell-based rules or hypotheses for an arbitrary number of signals (*e.g.* - contact with other cells, a substrate concentration, ECM density) increasing or decreasing a cell behavior (*e.g.* - cell speed, cell-cell adhesion, ECM production rate). The base form is as follows:

$$b(s) = b_0 + (b_{\mathrm{M}} - b_0) R(s) \tag{28}$$

where b_0 is the base level of a rate or other parameter modifiable at the cell level, b_{M} is the maximum quantity expected, and $R(s)$ is the functional relationship between the behavior b and signal s. In our examples, we use the default functional form for $R(s)$ - Hill functions. Each Hill function requires a half-maximum saturation value and Hill exponent. As more than one signal

may influence a behavior and the influence may be excitatory or inhibitory, Johnson et al. generalize the response of behavior b to a vector of $\mathbf{u}$ and $\mathbf{d}$ up and down signals respectively. These are used to produce the general form of Equation 28:

$$b(\mathbf{u}, \mathbf{d}) = (1 - D)\left((1 - U)b_0 + U \cdot b_{\mathrm{M}}\right) + D \cdot b_{\mathrm{M}} \tag{29}$$

where D and U are the total up and down responses to vectors of signals $\mathbf{u}$ and $\mathbf{d}$.

5 Model parameters

We performed a joint parameter sweep to determine the cell-cell adhesion and cell speeds using a preliminary version of the collective invasion model. Those initial values were modified for the additional example models. Similarly, the fiber realignment and reorientation rates were initially determined through single variable parameter sweep of preliminary versions of the collective invasion model. Parameters related to oxygen fields are from previous literature. Additional parameter values were selected to enable emergent behavior to occur at a time scale on the order of simulated days.

5.1 Invasive cellular front parameter details

See Tables 1 to 3 for the invasive cellular front parameters. Note there is no chemical environment or rules for this model.

**Parameter**	**Value**
Adhesion strength	0.4 μm/min
Repulsion strength	25.0 μm/min
Base cell speed	1.25 μm/min
ρ_{ideal}	0.5
ρ_{h}	1.0
ρ_{l}	0.0
ECM sensitivity	1.0
Rate of proliferation	0 min^{-1}
Rate of death	0 min^{-1}
Chemotactic bias	0.0
Persistence time	10 min
Fiber realignment rate (rate of change in anisotropy)	0.0 min^{-1}
Fiber reorientation rate	0.0 min^{-1}
Density modification rate	0.0 min^{-1}

Table 1: Cell-level parameters specific to the invasive cellular front model.

Parameter	Value
Initial anisotropy	1.0
Initial density	0.5
Initial orientation	random, horizontal, vertical, or mixed horizontal and vertical

Table 2: ECM initial conditions for invasive cellular front model.

Parameter	Value
Computational domain size	600 μm by 1000 μm
Number of cells added per cell addition step	30 cells
Cell addition step size	180 min

Table 3: Tissue level parameters for invasive cellular front model.

5.2 Fibrosis parameter details

See Tables 4 to 7 for the fibrosis model parameters. Rules are below.

Fibrosis cell rule details

In fibroblast cells:

- Contact with macrophage increases `custom:ECM_production_rate` (ECM density production) rate from 0.0001 min^{-1} towards 0.001 min^{-1} with a Hill response, with half-maximum 0.1 contacts and Hill power 10.

In dead cells:

- None

In macrophage cells:

- Contact with dead cell increases `inflammatory_signal` secretion from 0 towards 10 min^{-1} with a Hill response, with half-maximum 0.1 contacts and Hill power 10.
- Volume decreases `phagocytose dead cell` from 0.0005 min^{-1} towards 0.0001 min^{-1} with a Hill response, with half-maximum 2494 μm^3 and Hill power 10.

5.3 Basement membrane degradation parameter details

See Tables 8 to 11 for the invasive carcinoma parameters. Rules are below.

Basement membrane degradation rule details

In fibroblast cells:

- Contact with cancer cell decreases `custom:rules_based_speed_multiplier`, a dimensionless multiplier that reduces or increases base cell speed, from 1 towards 0.5 with a Hill response, with half-maximum 0.1 contacts and Hill power 4.

Parameter	Value
Adhesion strength	Fibroblast and macrophage - 5.0 μm/min, Dead cell - 10.0 μm/min
Repulsion strength	25.0 μm/min
Base cell speed	Fibroblast and macrophage - 0.5 μm/min, Dead cell - 0.0 μm/min
ρ_{ideal}	All - 0.5
ρ_{h}	All - 1.0
ρ_{l}	All - 0.0
ECM sensitivity	All - 0.0
Debris secretion rate	Dead cells - 1.0 min^{-1}
Debris target	Dead cells - 10
Debris uptake	Macrophages - 1.0 min^{-1}
Inflammatory signal secretion rate	Macrophages - see Fibrosis rules (Section 5.2)
Inflammatory signal target	Macrophages - 1.0
Rate of proliferation	All - 0 min^{-1}
Rate of death	All - 0 min^{-1} (dead cells begin the simulation already in a death process)
Transition rate from death state to removed from simulation	Dead cells - 0.001938 min^{-1}
Chemotactic bias	Fibroblasts and macrophages - 0.5
Persistence time	10 min
Fiber realignment rate (rate of change in anisotropy)	Fibroblast - 0.004 min^{-1}
Fiber reorientation rate	Fibroblast - 4 min^{-1}
ρ_{target}	Macrophages - 0.5, Fibroblast - 1.0
Density modification rate	Macrophages - 0.0 min^{-1}, Fibroblast - see Fibrosis rules (Section 5.2)

Table 4: Cell-level parameters specific to the fibrosis model.

Parameter	Value
Inflammatory signal diffusivity	1,000 μm^2/min
Inflammatory signal decay constant	1.0 min^{-1}
Debris diffusivity	1.0 μm^2/min
Debris decay constant	0.0 min^{-1}
Boundary condition type	No flux

Table 5: Biotransport and chemical microenvironment parameters for fibrosis model.

Parameter	Value
Initial anisotropy	0
Initial density	0.5
Initial orientation	random

Table 6: ECM initial conditions for fibrosis model.

Parameter	Value
Initial region of distressed/dead cell radius	175 μm
Computational domain size	1600 μm by 1600 μm
Pre-mechanics microenvironment conditioning duration	10 min

Table 7: Tissue level parameters for fibrosis model.

In cancer cells:

- contact with fibroblast decreases `inflammatory_signal` secretion from 50 min^{-1} towards 1 with a Hill response, with half-maximum 0.5 contacts and Hill power 4.
- Contact with fibroblast decreases `adhesive affinity to cancer cell` from 1 towards 0.25 with a Hill response, with half-max 0.1 and Hill power 4.
- Contact with fibroblast decreases `cell-cell adhesion` from 0.5 μm/min towards 0.25 μm/min with a Hill response, with half-max 0.1 contacts and Hill power 4.

**Parameter**	**Value**
Adhesion strength	Cancer cell - 0.5 μm/min, Fibroblast - see Invasive carcinoma rules (Section 5.3)
Repulsion strength	6.25 μm/min
Base cell speed	Cancer cell - 0.25 μm/min, Fibroblast - see Invasive carcinoma rules (Section 5.3)
ρ_{ideal}	Both - 0.5
ρ_{h}	Both - 1.0
ρ_{l}	Both - 0.0
Oxygen uptake	Both - 10 min^{-1} [2, 7]
ECM sensitivity	Cancer cell - 1.0, Fibroblast - 0.0
Inflammatory signal uptake	Fibroblast - 1.0 min^{-1}
Inflammatory signal secretion rate	Cancer cell - See Invasive carcinoma rules (Section 5.3)
Inflammatory signal target value	Cancer cell - 1.0
Rate of proliferation	Both - 0 min^{-1}
Rate of death	Both - 0 min^{-1}
Chemotactic bias	Fibroblasts - 0.95, cancer cells - 0.5
Persistence time	10 min
Fiber realignment rate (rate of change in anisotropy)	Fibroblast - 0.02 min^{-1}
Fiber reorientation rate	Fibroblast - 4 min^{-1}
ρ_{target}	Both - 0.5
Density modification rate	Cancer cell - 0 min^{-1}, Fibroblast - 0.001 min^{-1}

Table 8: Cell-level parameters specific to the basement membrane degradation model.

Parameter	Value
Oxygen diffusivity	100,000 μm^2/min [8]
Oxygen decay constant	0.1 min^{-1} [2, 7]
Oxygen boundary condition type	Mixed
Oxygen boundary condition value	38 mmHg oxygen (Dirichlet) [9], 0 mmHg/min^{-1}
Inflammatory signal diffusivity	1000 μm^2/min
Inflammatory signal decay constant	0.1 min^{-1}
Inflammatory signal boundary condition type	No flux

Table 9: Biotransport and chemical microenvironment parameters for basement membrane degradation model.

Parameter	Value
Initial anisotropy	0
Initial density	0.0 in lumen, 1.0 in basement membrane, 0.5 outside of duct
Initial orientation	random

Table 10: ECM initial conditions for the basement membrane degradation model.

Parameter	Value
Initial tumor radius	175 μm
Computational domain size	1600 μm by 1600 μm
Pre-mechanics microenvironment conditioning duration	10 min

Table 11: Tissue level parameters for basement membrane degradation model.

5.4 Collective migration parameter details

See Tables 12 to 15 for collective migration parameters. Note there are no cell rules for this model.

Remainder of page left blank on purpose.

Parameter	Value	Range
Adhesion strength	varies	0, 10 μm/min
Repulsion strength	25.0 μm/min	-
Base cell speed	varies	0.1, 0.5, 0.8 μm/min
ρ_{ideal}	0.5	-
ρ_{h}	1.0	-
ρ_{l}	0.0	-
ECM sensitivity	varies	0.0 (no reading), 1.0 (all other simulations), 0 for leaders (all simulations)
Oxygen uptake	10 min^{-1} [2, 7]	-
Rate of proliferation	0 min^{-1}	-
Rate of death	0 min^{-1}	-
Chemotactic bias	Leaders - 0.95, followers - dynamic	-
Persistence time	10 min	-
Fiber realignment rate (rate of change in anisotropy)	varies	0.0 (no writing), 0.001 (loss of collective migration scenario), 0.004 min^{-1}, 0 for followers (all simulations)
Fiber reorientation rate	varies	0.0 (no writing), 1 (loss of collective migration scenario), 4 min^{-1}, 0 for followers (all simulations)
ρ_{target}	0.5	-
Density modification rate	0.0	-

Table 12: Cell-level parameters specific to the collective migration model. Unless otherwise stated, parameters are at default values.

Parameter	Value
Oxygen diffusivity	100,000 μm^2/min [8]
Oxygen decay constant	0.1 min^{-1} [2, 7]
Boundary condition type	Dirichlet
Boundary condition value	38 mmHg oxygen [9]

Table 13: Biotransport and chemical microenvironment parameters for collective migration model.

Parameter	Value
Initial anisotropy	0
Initial density	0.5
Initial orientation	random

Table 14: ECM initial conditions for all collective migration simulations.

Parameter	Value
Initial organoid radius	175 μm
Computational domain size	1600 μm by 1600 μm
Pre-mechanics microenvironment conditioning duration	10 min
Cell fraction of leaders	0.05

Table 15: Tissue level parameters for collective migration model.

6 Links to supplementary videos

- Writing to ECM (local microstructure remodeling) - Cell-based ECM reorientation (Figure 2a)
- Reading ECM (contact guidance) - Circular ECM with no gradient (Figure 2b)
- Reading ECM (contact guidance) - Circular ECM with chemical gradient (Figure 2c)
- Invasive cell front - randomly oriented ECM (Figure 3)
- Invasive cell front - parallel-oriented ECM (Figure 3)
- Invasive cell front - perpendicular-oriented ECM (Figure 3)
- Invasive cell front - mixed ECM (Figure 3)
- Wound healing and fibrosis (Figure 4)
- Basement membrane degradation and transition of *in situ* carcinoma to invasion (Figure 5)
- Writing signals to ECM only (no reading/contact guidance) (Figure 6a)
- Reading signals from ECM only (no writing/microscale remodeling) (Figure 6b)
- Local microstructure remodeling (reading) and writing (contact guidance) leads to stigmergy (Figure 6c)
- Leader-follower - instant remodeling - higher cell speed (Figure 7a)
- Leader-follower - instant remodeling - medium cell speed (Figures 7b, 8a, SM4a)
- Leader-follower - instant remodeling - lower cell speed (Figure 7c)
- Collective migration - Non-instant signal writing (Figure 8b)
- Reading ECM (contact guidance) - split ECM pattern with chemical gradient (SM Figure 3)
- Collective migration - Loss of collective migration due to decreased remodeling rates (SM Figure 4b)

7 Supplementary Figures and invasive front results details

Scenario	Mean (μm)	Standard Deviation (μm)
Random	-124.4	12.5
Parallel	-162.0	0.0
Perpendicular	1.8	21.2

Table 16: Summary statistics of stochastic replicates of the invasive front simulation. We present the mean and standard deviation of the bin centers (y-coordinate) containing the 95th percentile of the cell count across all 21 replicates per scenario. For reference, the y-coordinates in this simulation runs from -500 μm to 500 μm.

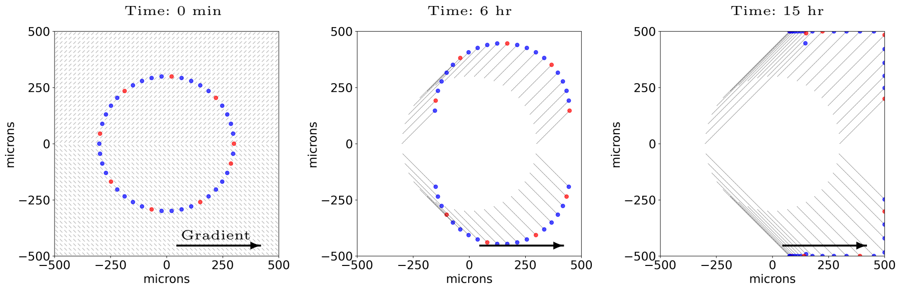

Supplementary Figure 3: **Highlight of individual aspects of the ECM-cell interactions: ECM orientation and cell chemotaxis** A combination of cues directing cell motility: ECM orientation (45° in the top of the domain and -45° in the bottom) and chemical gradient (to the right). The small black arrows trailing the cells show the positional history of each cell. The cell position is marked every six simulated minutes. Red and blue cells are identical; the coloring was added only for visual contrast. This is available as a video. See link the to video here.

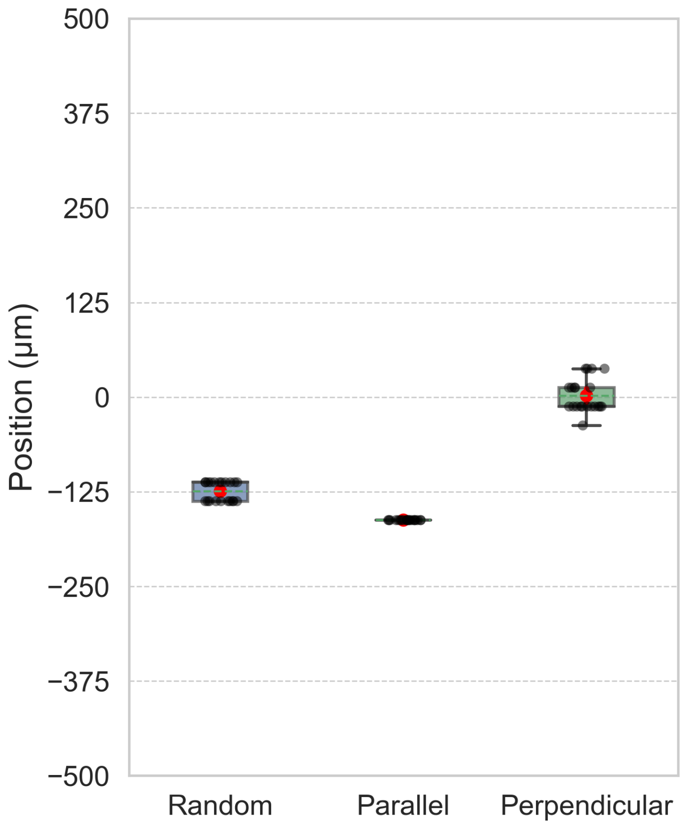

Supplementary Figure 4: **Extent of invasive front in invasive front scenarios:** Jitter and box and whisker plots of results across the random, parallel, and perpendicular ECM orientation scenarios. Each circle shows the y-position of the histogram bin containing the 95th percentile of cell count at five simulated days. The red circle marks each distributions' mean. Box and whiskers mark distribution quartiles.

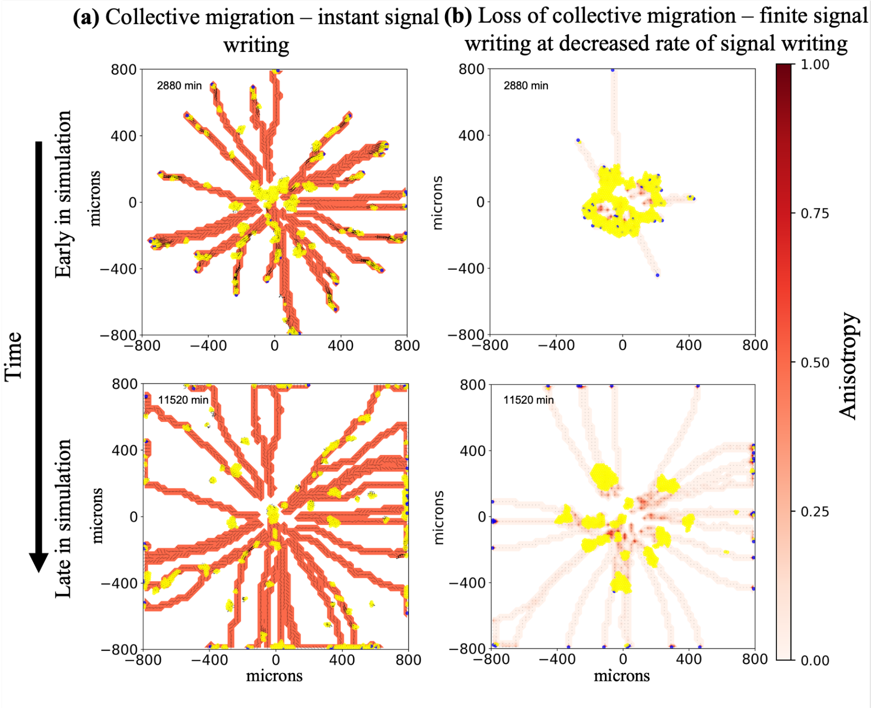

Supplementary Figure 5: **Collective migration requires sufficiently fast remodeling parameters:** Collective cell migration is observed in **(a)**, which has instantaneous ECM remodeling, whereas **(b)**, which has relaxed the instantaneous signal writing and slower rates of remodeling than those in Figure 8b of the main text, lacks collective migration. Fiber realignment rate: 0.001 min^{-1}, fiber reorientation rate: 1 min^{-1}. Videos are available. See the links here.

References

[1] Ghaffarizadeh, A., Heiland, R., Friedman, S.H., Mumenthaler, S.M., Macklin, P.: PhysiCell: An open source physics-based cell simulator for 3-D multicellular systems. PLOS Computational Biology **14**(2), 1005991 (2018). https://doi.org/10.1371/journal.pcbi.1005991

[2] Macklin, P., Edgerton, M.E., Thompson, A.M., Cristini, V.: Patient-calibrated agent-based modelling of ductal carcinoma in situ (DCIS): From microscopic measurements to macroscopic predictions of clinical progression. Journal of Theoretical Biology **301**, 122–140 (2012). https://doi.org/10.1016/j.jtbi.2012.02.002

[3] D'Antonio, G., Macklin, P., Preziosi, L.: An agent-based model for elasto-plastic mechanical interactions between cells, basement membrane and extracellular matrix. Mathematical Biosciences and Engineering **10**(1), 75–101 (2013). https://doi.org/10.3934/mbe.2013.10.75

[4] Drasdo, D., Kree, R., McCaskill, J.S.: Monte Carlo approach to tissue-cell populations. Physical Review E **52**(6), 6635–6657 (1995). https://doi.org/10.1103/PhysRevE.52.6635

[5] Galle, J., Loeffler, M., Drasdo, D.: Modeling the Effect of Deregulated Proliferation and Apoptosis on the Growth Dynamics of Epithelial Cell Populations In Vitro. Biophysical Journal **88**(1), 62–75 (2005). https://doi.org/10.1529/biophysj.104.041459

[6] Johnson, J.A.I., Stein-O'Brien, G.L., Booth, M., Heiland, R., Kurtoglu, F., Bergman, D.R., Bucher, E., Deshpande, A., Forjaz, A., Getz, M., Godet, I., Lyman, M., Metzcar, J., Mitchell, J., Raddatz, A., Rocha, H., Solorzano, J., Sundus, A., Wang, Y., Gilkes, D., Kagohara, L.T., Kiemen, A.L., Thompson, E.D., Wirtz, D., Wu, P.-H., Zaidi, N., Zheng, L., Zimmerman, J.W., Jaffee, E.M., Chang, Y.H., Coussens, L.M., Gray, J.W., Heiser, L.M., Fertig, E.J., Macklin, P.: Digitize Your Biology! Modeling Multicellular Systems through Interpretable Cell Behavior. bioRxiv (2023). https://doi.org/10.1101/2023.09.17.557982

[7] Ghaffarizadeh, A., Friedman, S.H., Macklin, P.: BioFVM: An efficient, parallelized diffusive transport solver for 3-D biological simulations. Bioinformatics **32**(8), 1256–1258 (2016). https://doi.org/10.1093/bioinformatics/btv730

[8] Grote, J., Süsskind, R., Vaupel, P.: Oxygen diffusivity in tumor tissue (DS-Carcinosarcoma) under temperature conditions within the range of 20–40°C. Pflügers Archiv **372**(1), 37–42 (1977). https://doi.org/10.1007/BF00582204

[9] McKeown, S.R.: Defining normoxia, physoxia and hypoxia in tumours—implications for treatment response. The British Journal of Radiology **87**(1035), 20130676 (2014). https://doi.org/10.1259/bjr.20130676
